# Structure and function of the RAD51B-RAD51C-RAD51D-XRCC2 tumour suppressor

**DOI:** 10.1038/s41586-023-06179-1

**Published:** 2023-06-21

**Authors:** Luke A. Greenhough, Chih-Chao Liang, Ondrej Belan, Simone Kunzelmann, Sarah Maslen, Monica C. Rodrigo-Brenni, Roopesh Anand, Mark Skehel, Simon J. Boulton, Stephen C. West

**Affiliations:** 1 The Francis Crick Institute, 1 Midland Road, London NW1 1AT, U.K.

## Abstract

Homologous recombination is a fundamental process of life. It is required for the protection and restart of broken replication forks, the repair of chromosome breaks, and the exchange of genetic material during meiosis. Individuals with mutations in key recombination genes, such as *BRCA2 (FANCD1)*, or the *RAD51* paralogs (*RAD51B, RAD51C [FANCO], RAD51D, XRCC2* [*FANCU*] and *XRCC3*) are predisposed to breast, ovarian and prostate cancers^1–10^, or the cancer prone syndrome Fanconi anemia^11–13^. The product of *BRCA2*, the BRCA2 tumour suppressor protein, is well characterised, but the cellular functions of the RAD51 paralogs are unclear. Gene knockouts display growth defects, reduced RAD51 focus formation, spontaneous chromosome abnormalities, sensitivity to PARP inhibitors and replication fork defects^14,15^, but their precise molecular roles in fork stability, DNA repair and cancer avoidance remain unknown. Here, we used cryo-electron microscopy, AlphaFold2 modelling and structural proteomics to define the high-resolution structure of the RAD51B-RAD51C-RAD51D-XRCC2 (BCDX2) complex, revealing that RAD51C-RAD51D-XRCC2 mimic three RAD51 protomers aligned within a nucleoprotein filament, whereas RAD51B is highly dynamic. Biochemical and single-molecule analyses showed that BCDX2 stimulates the nucleation and extension of RAD51 filaments, which are essential for recombinational DNA repair, in reactions dependent on the coupled ATPase activities of RAD51B and RAD51C. Our studies demonstrate that BCDX2 orchestrates RAD51 assembly on single-stranded DNA for replication fork protection and double strand break repair, in reactions that are critical for tumour avoidance.

RecA/RAD51 recombinases are highly conserved throughout all species^16^. Recombinational repair of DNA double strand breaks requires the formation of helical nucleoprotein filaments of RAD51 on resected RPA-coated single stranded DNA (ssDNA) termini, that promote homologous pairing and strand exchange with the sister chromatid^17–23^. Filament formation extends DNA to 1.5x the length of B-form DNA (pitch approx. 100 Å, 6 RecA/RAD51 protomers per helical turn) and is dependent upon ATP binding. While bacterial RecA forms filaments efficiently, eukaryotic RAD51’s need auxiliary factors to stimulate their interaction with ssDNA^24^. In human cells, filament assembly is driven by a nucleation-extension mechanism and requires BRCA2, PALB2 and the RAD51 paralogs. BRCA2 binds RAD51 via eight conserved BRC repeats, stabilising RAD51 filaments via a C-terminal RAD51 binding region and binding directly to ssDNA via OB-folds^25–33^. PALB2, a chromatin associated protein, links BRCA2 to upstream signalling BRCA1 and helps guide RAD51 to sites of DNA damage^34–36^. In contrast, the precise role of the five RAD51 paralogs is unknown. The proteins assemble into two distinct complexes, with RAD51C mutual to both: RAD51B-RAD51C-RAD51D-XRCC2 (BCDX2) and RAD51C-XRCC3 (CX3)^37^, but molecular analyses of their structure and function have been hindered by insolubility issues. Instead, efforts have focused on related complexes from *S. cerevisiae* (Rad55-Rad57) or *C. elegans* (Rfs-1/Rip-1), which promote RAD51 filament growth by the transient capping of filament ends^38–40^.

## Structure of the BCDX2 complex

**Extended Data Fig. 1:**
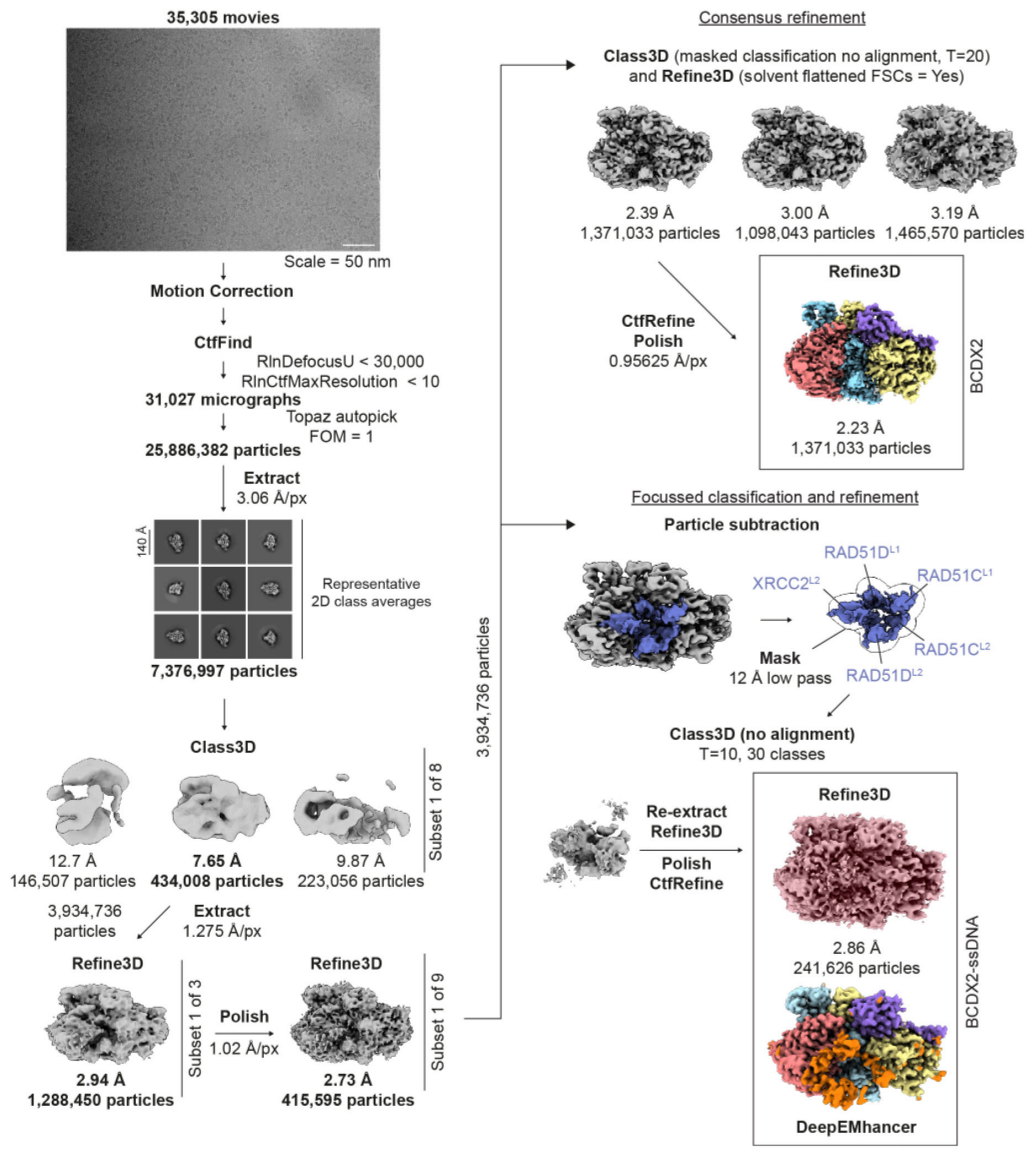
Single particle analysis pipeline of BCDX2-ADP.AlFx and BCDX2-ADP.AlFx-ssDNA. Summary of the data processing strategies, including intermediate 2D and 3D class averages, which yield the final reconstructions of BCDX2-ADP.AlFx (2.2 Å) and BCDX2-ADP.AlFx-ssDNA (2.9 Å).

**Extended Data Fig. 2:**
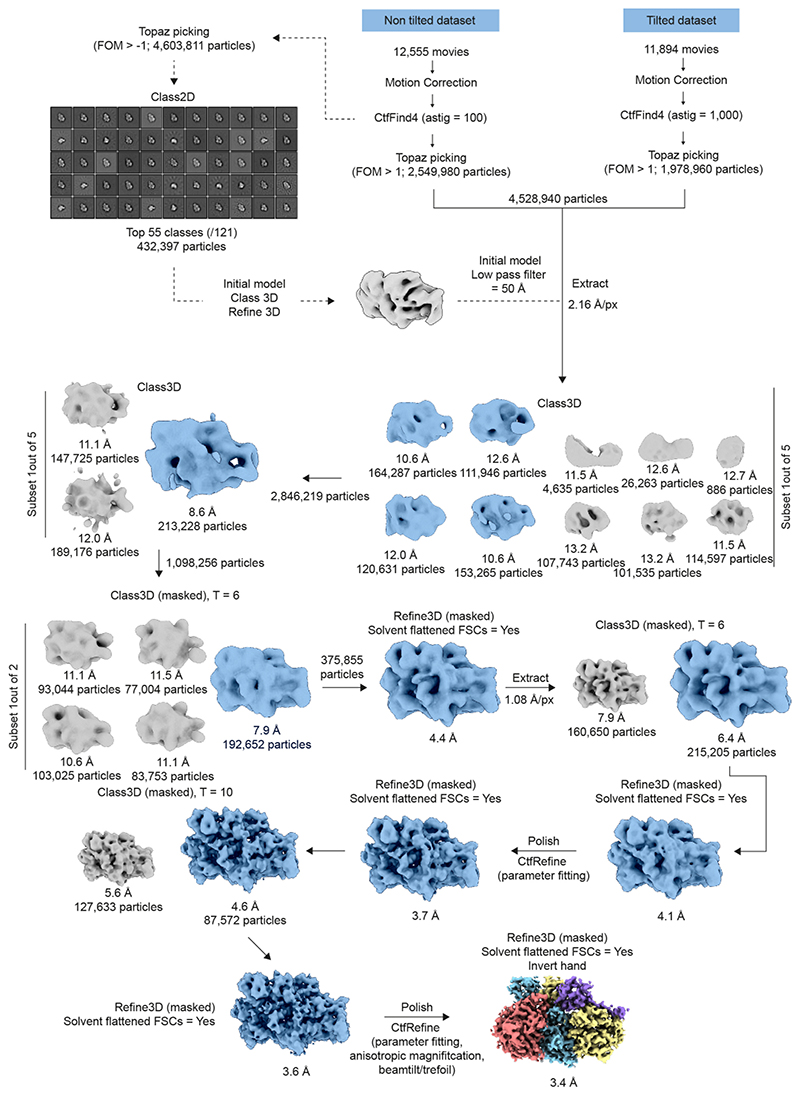
Single particle analysis pipeline of BCDX2-ADP.BeFx. Summary of the data processing strategy, including intermediate 2D and 3D class averages, which yields the final reconstruction of BCDX2-ADP.BeFx (3.4 Å)

**Extended Data Fig. 3:**
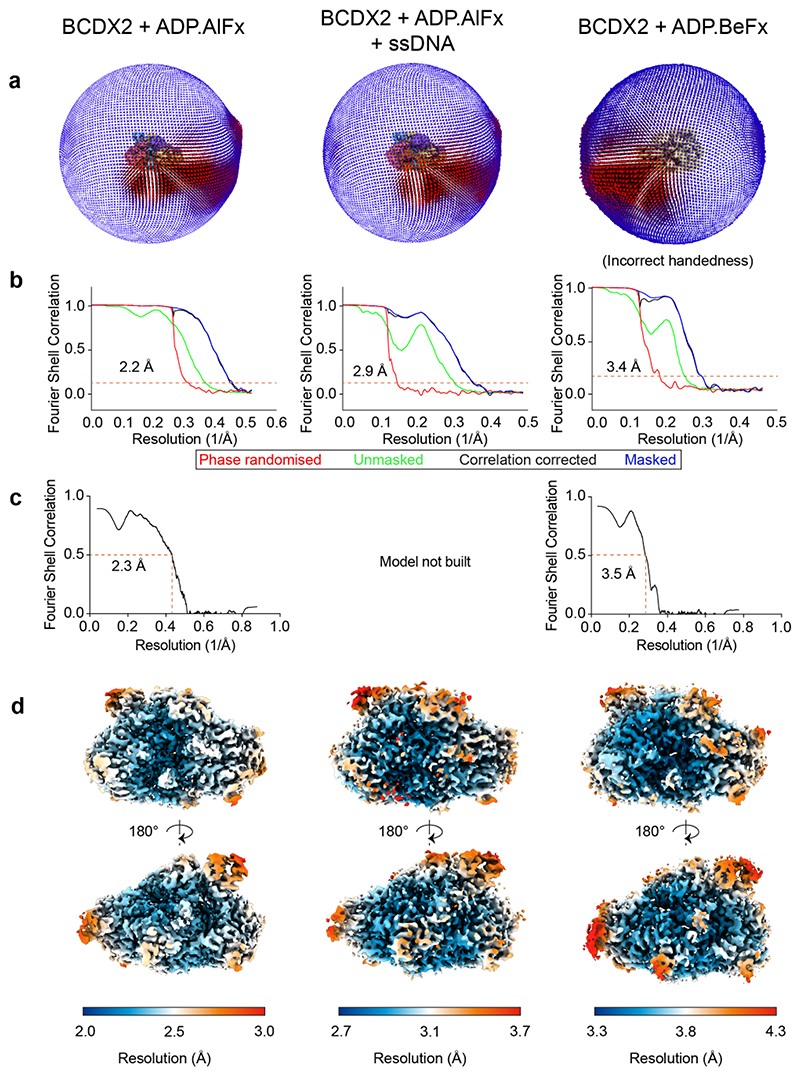
Angular distribution, map and model resolution statistics and local resolution. **a**, Angular distribution plots. **b**, Fourier Shell Correlation (FSC) plots. **c**, Map vs model FSCs. **d**, Local resolution estimates for BCDX2-ADP.AlFx (2.2 Å), BCDX2- ADP.AlFx (2.9 Å) and BCDX2-ADP.BeFx (3.4 Å).

**Extended Data Fig. 4:**
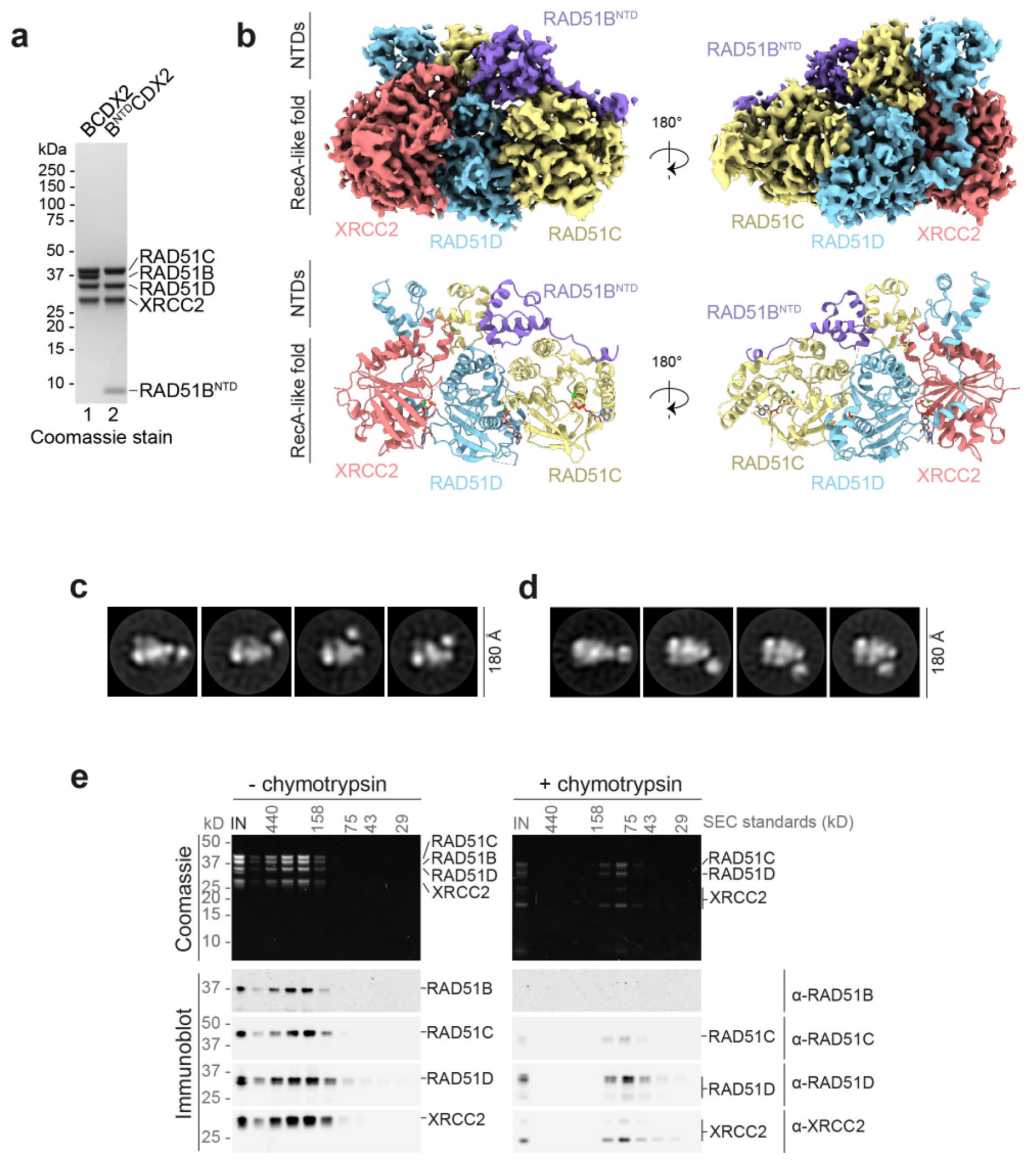
Structural and biochemical analyses of BCDX2. **a,** SDS-PAGE of BCDX2 and B^NTD^CDX2. **b**, Front (left) and back (right) views of BCDX2-ADP.BeFx cryo-EM map (3.4 Å) and atomic model. **c**, and **d**, NS-EM 2D class averages of BCDX2 showing movement of the mobile domain in the presence of ATP or ADP, respectively. **e**, Limited proteolysis of BCDX2. SDS-PAGE gel and immunoblots of Superdex 200 3.2/300 fractions for untreated and chymotrypsin treated BCDX2. For gel and immunoblot source data, see Supplementary Figure 1.

**Fig. 1:**
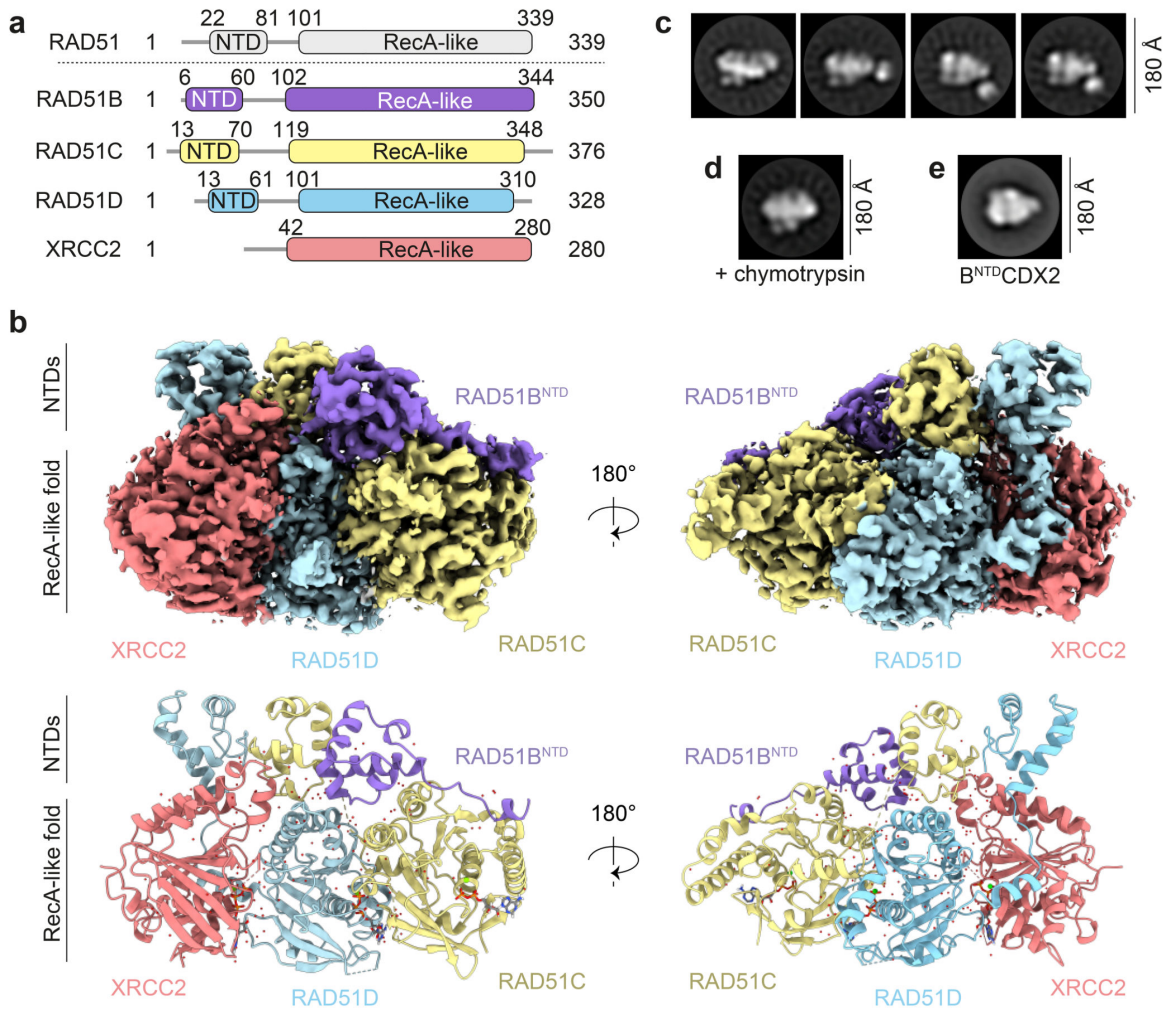
Cryo-EM structure of BCDX2. **a,** Domain architectures of RAD51, RAD51B, RAD51C, RAD51D and XRCC2 protein subunits. NTD = N-terminal domain. **b,** Front and back views of the cryo-EM map (2.2 Å) and atomic model of BCDX2 in the presence of ADP.AlFx. **c,** Representative NS-EM 2D class averages of BCDX2 showing a mobile domain relative to the structural core. **d,** Representative 2D class average of chymotrypsin treated BCDX2. **e**, Recombinant B^NTD^CDX2 deleted for the RAD51B^CTD^.

The human RAD51 paralogs have conserved RecA-like folds containing three nucleotide-binding motifs, the Walker A and B motifs, and a ‘lysine finger’ ATP cap, in addition to the L1 and L2 DNA binding loops^41^. RAD51, RAD51B, RAD51C and RAD51D also share a conserved N-terminal domain (NTD) α-helical bundle, which is absent in XRCC2 (Fig. 1a). To identify the molecular functions of human BCDX2, we determined its structure using cryo-electron microscopy (cryo-EM). BCDX2 was purified to homogeneity following expression in baculovirus-infected insect cells, and high-resolution structures were obtained in the presence of transition and ground state analogs of ATP, ADP.AlFx (2.2 Å) and ADP.BeFx (3.4 Å) (Fig. 1b, Extended Data Figs. 1, 2, 3 and 4a, b, Extended Data Table 1).

In atomic models, the three NTDs of RAD51B, RAD51C and RAD51D, and three out of the four C-terminal RecA-like folds (RAD51C, RAD51D and XRCC2) were observed. We were unable to resolve cryo-EM density for the C-terminal RecA-like fold of RAD51B (Fig. 1b), but negative stain electron microscopy (NS-EM) 2D class averages revealed a mobile feature relative to a structured core (Fig. 1c), irrespective of the nucleotide present (Extended Data Fig. 4c, d). Following limited proteolysis with chymotrypsin, RAD51B could no longer be detected by Western blotting, coincident with an increased retention time in gel filtration and a loss of the mobile density in NS-EM 2D class averages. These results show that the mobile density corresponds to the RAD51B C-terminal domain (CTD) (Fig. 1d, Extended Data Fig. 4e). Confirmation was obtained by NS-EM 2D classification of purified BCDX2 from which the RAD51B^CTD^ had been deleted (B^NTD^CDX2) (Fig. 1e, Extended Data Fig. 4a1). Thus, the C-terminal RecA-like fold of RAD51B is dynamic and RAD51B’s interaction with RAD51C is driven primarily through the RAD51B^NTD^ and linker.

**Fig. 2:**
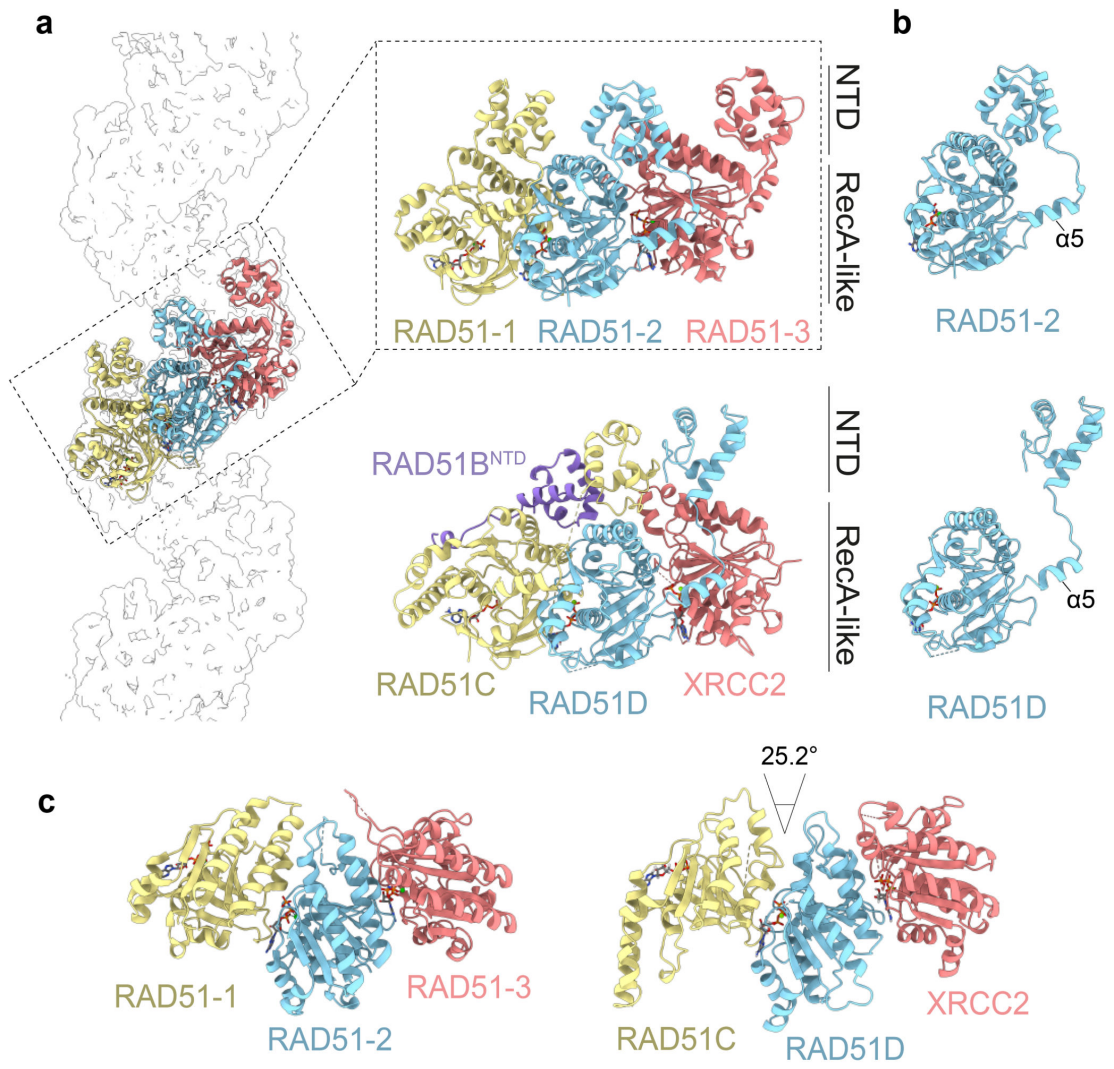
Structural comparison between BCDX2 and RAD51 filaments. **a**, Zoomed view of three RAD51 protomers (RAD51-1, RAD51-2, RAD51-3) (PDB:5H1B) aligned relative to RAD51B^NTD^-RAD51C-RAD51D-XRCC2. **b**, Comparison of the NTD-linker-α5 in RAD51-2 and RAD51D. **c**, Top views of the C-terminal RecA-like folds of three RAD51 protomers and RAD51C-RAD51D-XRCC2 subunits. The 25.2° anticlockwise rotation of RAD51C relative to RAD51-1 is indicated.

The BCDX2 atomic model revealed striking similarities between the arrangement of the RecA-like domains in RAD51C-RAD51D-XRCC2 and three RAD51 protomers assembled in a nucleoprotein filament (Fig. 2a), by the stacking of the RecA-like folds and nucleotides bridging subunit interfaces. As with RAD51, the linker and helix (α5) that tethers the NTD to the RecA-like fold of each subunit makes key contacts with the adjacent subunit^42^. However, in the RAD51 filament, the NTD of one protomer binds the same protomer (in *cis*), whereas in BCDX2 the NTD of one subunit binds the adjacent subunit (in *trans*) (Fig. 2a, b). Moreover, the three NTDs of RAD51B, RAD51C and RAD51D are tightly stacked together, in contrast to both RAD51 and RecA where they are spatially separated to create an entry point for the invading duplex DNA^43^. The tight clustering of NTDs drives further specificity for paralog-paralog interactions, whereas engagement of the RAD51B^NTD^ *in trans* ensures the dynamic association of RAD51B with the structural core. Finally, the alignment of the RecA-like folds of RAD51C-RAD51D-XRCC2 relative to three RAD51 protomers within a filament shows an open configuration (25.2° decreased curvature) (Fig. 2c), in contrast with RAD51 where tight protomer contacts provide the high degree of curvature required for helical filament formation.

**Fig. 3:**
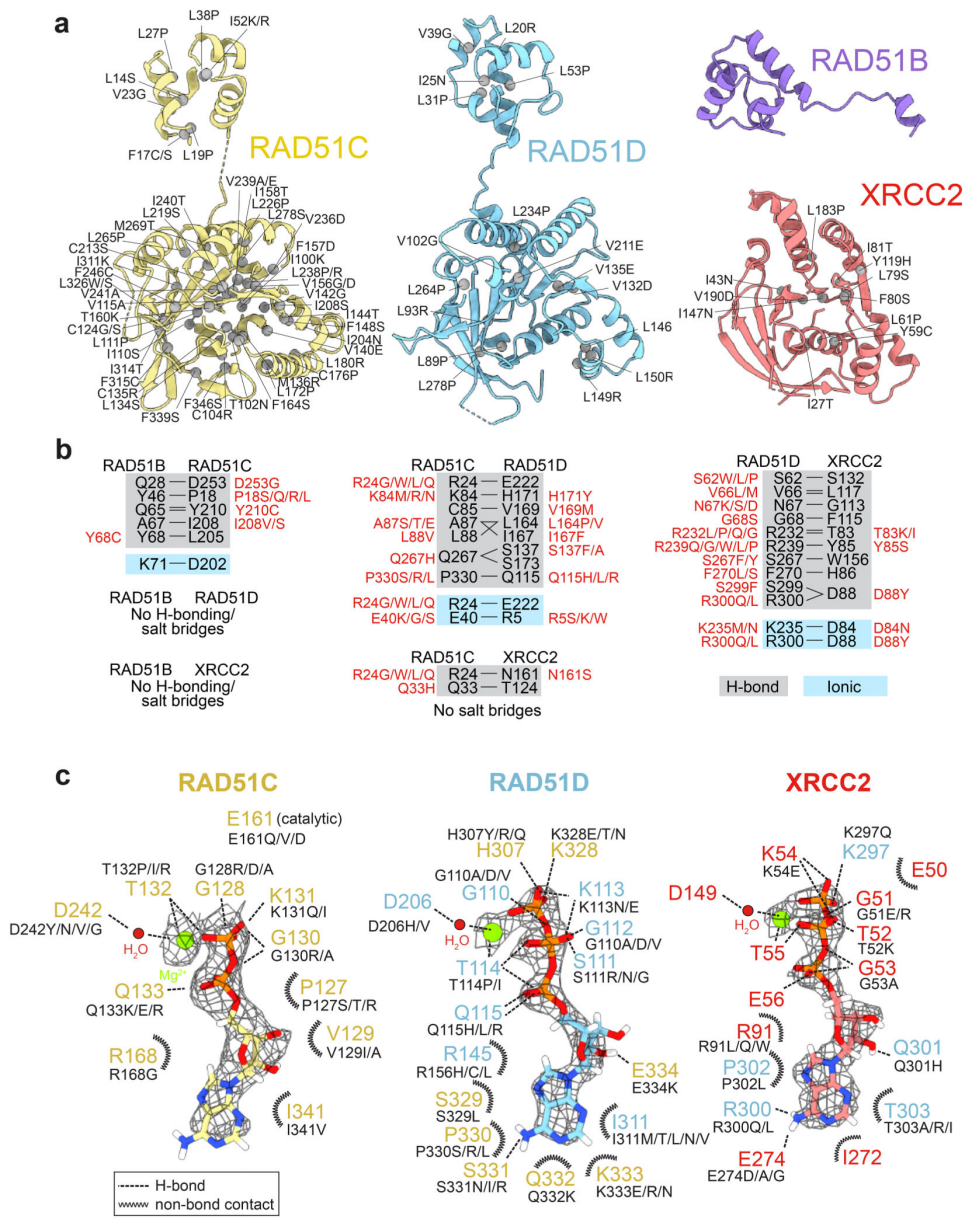
Structural insights into *RAD51B, RAD51C, RAD51D* and *XRCC2* VUS. Missense VUS mutations in the ClinVar database were extracted for RAD51B (21), RAD51C (811), RAD51D (689) and XRCC2 (283). **a**, *In silico* prediction of missense mutations which affect monomer stability of RAD51B, RAD51C, RAD51D or XRCC2. **b**, Hydrogen and ionic bonds between RAD51 paralogs, and associated VUS. **c**, Interaction diagram of hydrogen bonds and non-bonded contacts in the nucleotide binding sites of RAD51C, RAD51D and XRCC2. Density of the nucleotides and Mg^2+^ is shown as black mesh. Stratification of ClinVar mutations highlighted in Supplementary Table 1.

Mutations in *RAD51B, RAD51C, RAD51D* and *XRCC2* are known to cause hereditary breast/ovarian cancer and Fanconi anemia. We therefore extracted all reported pathogenic and variants of uncertain significance (VUS) missense mutations from the ClinVar database^44^ and explored potential pathogenicity by analysing how they might affect subunit stability, complex formation, nucleotide binding/hydrolysis and DNA binding. Missense mutations likely to disrupt monomer folding of RAD51C, RAD51D and XRCC2 are indicated (Fig. 3a), as are mutations in residues that could affect hydrogen and ionic bonding at the subunit-subunit interfaces (Fig. 3b). We also observed that many mutations cluster around the nucleotide binding sites of RAD51C, RAD51D and XRCC2 (Fig. 3c). All variants and their stratification are highlighted in Supplementary Data Table 1.

## RAD51B and RAD51C ATPases are coupled

**Extended Data Fig. 5:**
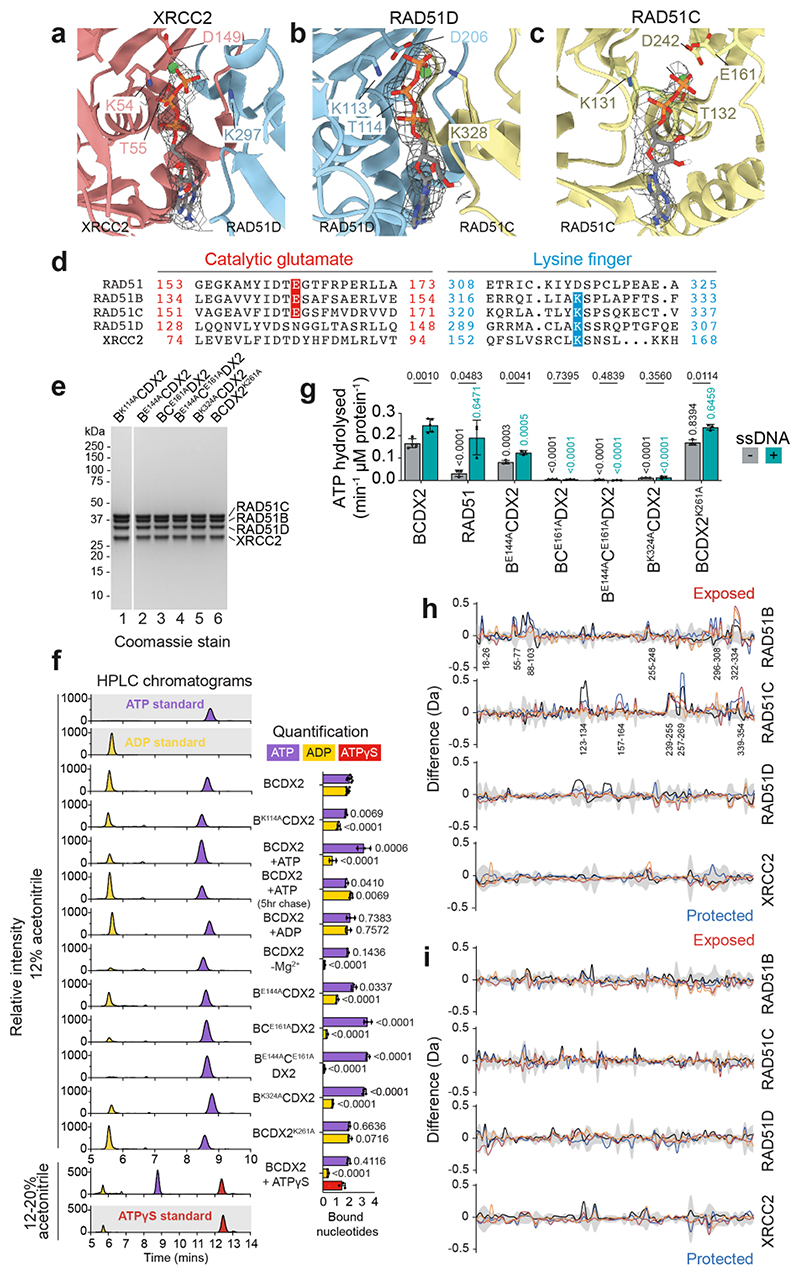
Coupling of RAD51B and RAD51C ATPases. **a**, **b** and **c**, Atomic models from the BCDX2-ADP.BeFx structure (3.4 Å) showing the binding of ATP by XRCC2, ATP by RAD51D and ADP by RAD51C, respectively. The Walker A lysine and threonine, Walker B aspartate, catalytic glutamate and the lysine finger from the adjacent subunit are indicated. Density of the nucleotide is shown as black mesh. Green spheres = Mg^2+^ ions. **d**, Sequence alignment of the RAD51, RAD51B, RAD51C, RAD51D and XRCC2 protein sequences. Highlighted in red are conserved catalytic glutamate residues (RAD51, RAD51B and RAD51C) and in cyan are lysine fingers (RAD51B, RAD51C, RAD51D and XRCC2). **e**, SDS-PAGE of the mutant proteins used in Figure 4. For gel source data, see Supplementary Figure 1. **f,** HPLC chromatograms (left) of standards (standardised to 2 μM ATP, ADP, ATPyS) and BCDX2/B^CTD^ (standardised to 1 μM BCDX2). Bar chart (mean + s.d.) of bound nucleotides (right). All are n=3 except wt and B^K114A^CDX2 which is n=7 and n=4 respectively. n values are independent experiments. Unpaired two-tailed t-test. **g**, Bar chart (mean + s.d.) of ATP hydrolysis rate. All are n=3 except wt which is n=6. n values are independent experiments. Unpaired two-tailed t-test. **h** and **i**, Difference plots between BCDX2-ADP.AlFx and BCDX2-ADP, and BCDX2-ADP.BeFx and BCDX2-ADP, respectively, showing the level of deuterium uptake after 3 (orange), 30 (red), 300 (blue) and 3000 (black) seconds. Positive values = exposure; negative values = protection. RAD51B: 116 peptides, 91.1% coverage, 3.36 redundancy. RAD51C: 134 peptides, 88% coverage, 4.11 redundancy. RAD51D: 99 peptides, 93.0% coverage, 3.5 redundancy. XRCC2: 76 peptides, 93.6% coverage, 2.67 redundancy.

**Fig. 4:**
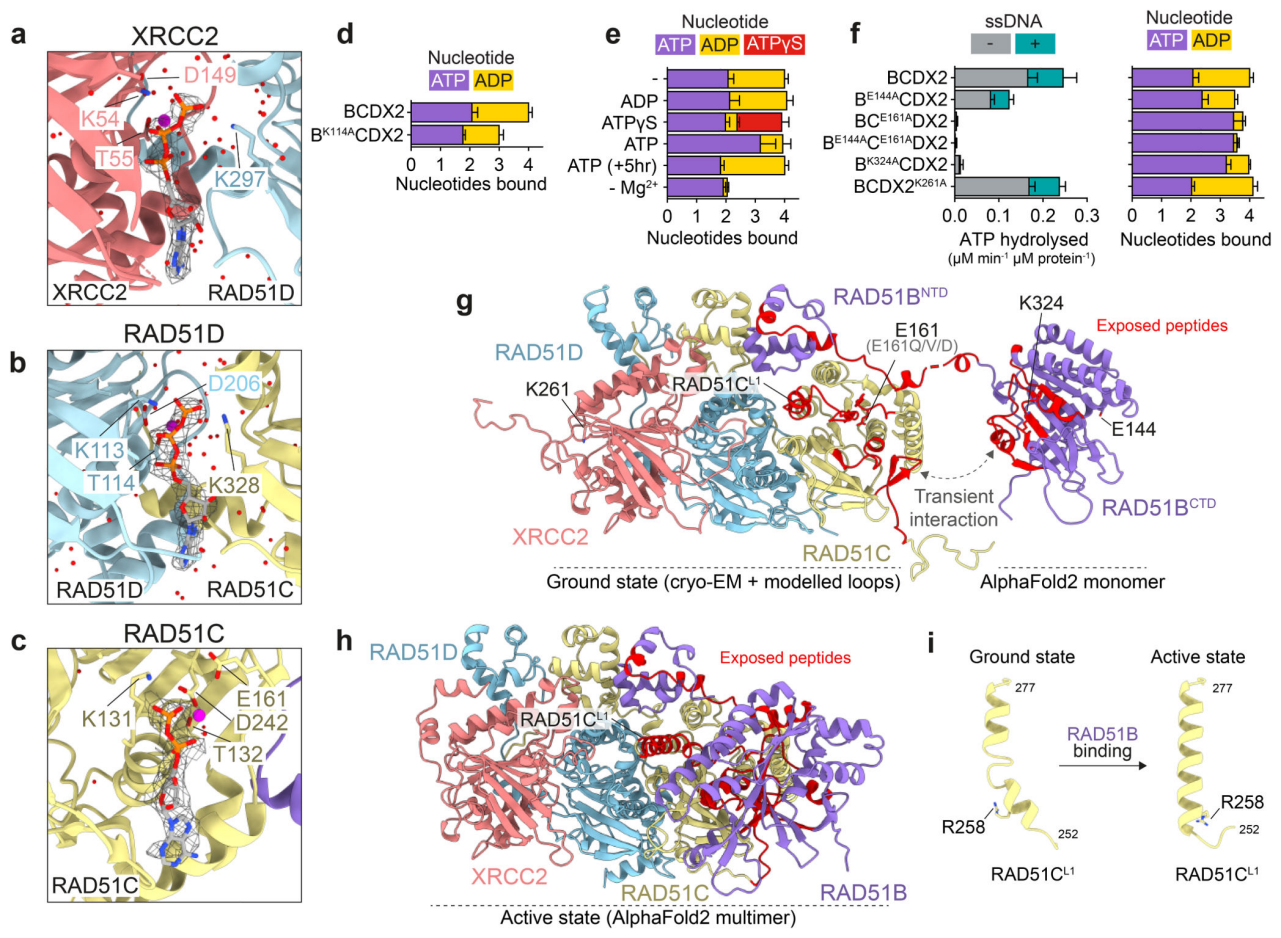
Coupled ATPase activities of RAD51B and RAD51C. **a**, **b**, and **c**, Atomic models from the BCDX2-ADP.AlFx structure (2.2 Å) showing the binding of ATP by XRCC2 and RAD51D, and ADP by RAD51C. The Walker A lysine and threonine, Walker B aspartate, Mg^2+^ cations (magenta spheres) and water molecules (red dots) are indicated. Lysine fingers RAD51D^K297^ and RAD51C^K308^ that coordinate the XRCC2 and RAD51D active sites (respectively) and the catalytic glutamate RAD51C^E161^ are shown. Density of the nucleotide is shown as black mesh. **d**, Quantification of nucleotides bound by BCDX2 (n=7) and B^K114A^CDX2 (n=4) as measured by HPLC analysis. Mean + s.d. **e**, Quantification of nucleotides bound by BCDX2 following exchange with no nucleotide (n=7), ADP (n=3), ATP yS (n=3), or ATP (n=3). ATP (+5 hr) are repeat measurements of the ATP sample after 5 hours at room temperature (n=3). -Mg^2+^ is BCDX2 in the absence of magnesium cations (n=3). Mean + s.d. n values represent independent experiments. **f**, Quantification of ATPase activity (left) and nucleotides bound (right) for BCDX2 (ATPase: n=6; bound nucleotides = n=7), B^E144A^CDX2 (n=3), BC^E161A^DX2 (n=3), B^E144A^C^E161A^DX2 (n=3), B^K324A^CDX2 (n=3) and BCDX2^K261A^ (n=3). Mean + s.d. n values represent independent experiments. **g**, Comparative HDX-MS analysis of BCDX2-ADP.AlFx vs BCDX2-ADP. Regions of exposure during AlFx release are indicated (red). Hybrid model contains cryo-EM determined atomic model of B^NTD^CDX2 with missing loops modelled, together with the AlphaFold2-predicted monomer model of RAD51B^CTD^. The catalytic glutamate residues RAD51B^E144^ and RAD51C^E161^, and lysine fingers RAD51B^K324^ and XRCC2^K261^, are highlighted. **h**, As (g) but mapped onto the AlphaFold2 multimer model of BCDX2. **i**, Extension of the RAD51C^L1^ helix and rotation of RAD51C^R258^ during RAD51C ATP hydrolysis and RAD51B binding. All statistics associated with this figure are found in Extended Data Fig. 5.

For the cryo-EM analyses, BCDX2 was vitrified in the presence of ADP.AlFx or ADP.BeFx. However, we did not observe density for AlFx or BeFx in either cryo-EM map, and instead observed that RAD51D and XRCC2 bound ATP whereas RAD51C bound ADP (Fig. 4a-c, Extended Data Fig. 5a-c). The ATP remained bound to BCDX2 during protein purification. RAD51D’s key coordination points with ATP included hydrogen bonding of Walker A K113 with β- and γ-phosphates, and Walker A T114 and Walker B D206 residues (the latter via an ordered water molecule) interacting with a Mg^2+^ ion bridging the β- and γ-phosphates. XRCC2 binds ATP in a near-identical arrangement via Walker A K54/T55 and Walker B D149. In contrast, RAD51C bound ADP through Walker A K131, T132 and Walker B D242 residues. Although the arrangement of bound nucleotides are similar to those found in RecA/RAD51, cryo-EM analyses failed to: (i) explain the presence of ATP in the active sites of RAD51D and XRCC2 given that samples were vitrified with ADP.AlFx/BeFx, or (ii) identify the nucleotide bound by RAD51B. To address these points, we extracted nucleotides (nt) from purified BCDX2 complexes and analysed their composition by HPLC (Fig. 4d), finding that BCDX2 bound 4 nt (2.1 ± 0.2 nt of ATP; 1.9 ± 0.1 nt of ADP) whereas the B^K114A^CDX2 Walker A mutant bound only 3 nt (ATP = 1.8 ± 0.1 nt; ADP = 1.2 ± 0.1). These results show that ADP occupies the RAD51B and RAD51C nucleotide binding sites. Nucleotide exchange experiments, in which BCDX2 was incubated with ADP, ATPγS or ATP, revealed that addition of ADP did not affect the ATP/ADP ratio (Fig. 4e), showing that the ATPs bound by RAD51D and XRCC2 are non-exchangeable. In contrast, the ADP bound by RAD51B or RAD51C was replaced by ATPγS or ATP. Removal of Mg^2+^ cations led to a loss of bound ADP, confirming the presence of Mg^2+^ in the atomic model of RAD51C’s active site and that the nucleotides bound to RAD51B and RAD51C are labile.

The catalytic glutamate residue of RAD51 (RAD51^E163^) that is required for cleavage of the covalent β-γ phosphate bond of ATP is conserved in RAD51B (E144) and RAD51C (E161), and RAD51C^E161^ mutations have been linked to breast/ovarian cancers (Fig. 3c). These glutamates are not conserved in RAD51D or XRCC2 explaining their inability to promote ATP hydrolysis while retaining nucleotide binding. Other key residues are the ‘lysine fingers’, which trans-coordinate the γ-phosphate of the adjacent subunit and are observed in all four RAD51 paralogs, but not RAD51 itself (Extended Data Fig. 5d). Two lysine fingers, RAD51D^K297^ and RAD51C^K328^, coordinate ATP in the XRCC2 and RAD51D nucleotide binding sites (Fig. 4a, b), whereas the functions of RAD51 B^K324^ and XRCC2^K261^ are unknown.

To dissect the ATPase catalytic cycle of BCDX2, catalytic glutamate mutants of RAD51B and RAD51C (B^E144A^CDX2, BC^E161A^DX2, B^E144A^C^E161A^DX2), and lysine finger mutants of RAD51B and XRCC2 (B^K324A^CDX2 and BCDX2^K261A^) were purified. A 50% reduction in the ATPase activity of B^E144A^CDX2 relative to wild-type was observed, coincident with an increased fraction of ATP present within the complex (Fig. 4f). Surprisingly, mutation of RAD51C (BC^E161A^DX2 and B^E144A^C^E161A^DX2) resulted in a complete loss of ATPase activity, such that all nucleotide binding sites were fully occupied with ATP. These results show that the ATPase activities of RAD51B are dependent on RAD51C. We also found that the lysine finger mutant B^K324A^CDX2 exhibited reduced ATP hydrolysis whereas the equivalent mutation in XRCC2 (BCDX2^K261A^) did not affect ATPase activity (Fig. 4f). SDS-PAGE of all BCDX2 mutants, HPLC chromatograms, quantification and statistics, and ATPase quantification and statistics are found in Extended Data Fig. 5e-g.

Hydrogen-deuterium exchange mass spectrometry (HDX-MS), in the presence of ADP.AlFx or ADP, was used to detect conformational changes that occur during the ATPase catalytic cycle. We observed increased deuterium uptake (i.e., additional solvent exposure) on the RAD51C L1 DNA binding loop, the nucleotide-binding pocket of RAD51C, the surface of RAD51B containing the lysine finger K324, and the NTD-CTD linker following γ-phosphate (AlFx) release (Fig. 4g, Extended Data Fig. 5h). These data indicate that RAD51B^K324^ engages with RAD51C during ATP hydrolysis, and that RAD51B’s ATPase is allosterically activated. Conformational changes were not observed in comparative HDX-MS experiments of ADP.BeFx with ADP (Extended Data Fig. 5i), indicating that interactions between RAD51B^CTD^ and RAD51C^CTD^ are stabilised through the ATP transition (ADP.AlFx) as opposed to the pre-hydrolytic (ADP.BeFx) state^45^.

AlphaFold2-multimer predictions of BCDX2^7,46^ indicate direct interactions between RAD51B^CTD^ and RAD51C^CTD^, consistent with the conformational changes identified with HDX-MS (Fig. 4h). The overall structure of B^NTD^CDX2 remained the same, with one exception being the extension of the helix proceeding the L1 DNA binding loop, resulting in the rotation of RAD51C , a residue that is mutated to histidine in 1 Q Fanconi anaemia patients’^12^ (Fig. 4i). We therefore suggest that the Alphafold2 predicted structure corresponds to the active intermediate during the ATP hydrolytic cycle, whereas the cryo-EM structures represent the ground state of BCDX2.

## BCDX2 promotes RAD51 filament formation

**Extended Data Fig. 6:**
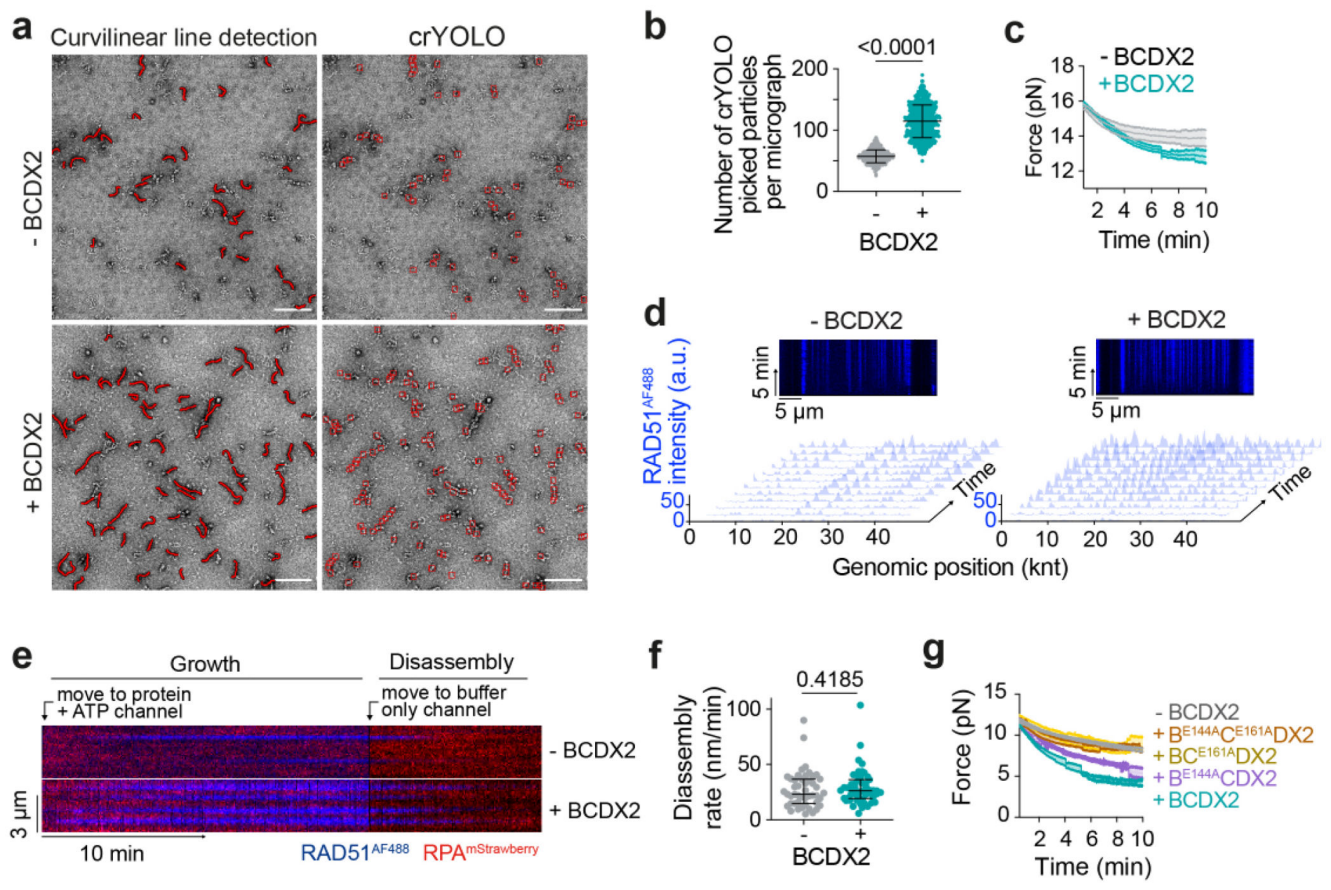
ATP hydrolysis by BCDX2 stimulates RAD51 filament assembly. **a,** Representative NS-EM micrographs of RAD51 filaments in the absence and presence of BCDX2, showing filaments detected by curvilinear line analysis and crYOLO particle picking. Scale bar = 100 nm. **b**, Scatter plot (mean + s.d.) of number of crYOLO picked particles per micrograph (n=631 micrographs). Unpaired two-tailed t-test. **c**, Force measured between the traps as a function of time in the absence (n=6) and presence (n=7) of BCDX2. Shaded area represents SEM. **d**, Representative kymographs and time-binned intensity histograms verses genomic position on RPA^mStrawberry^ coated λ ssDNA for RAD51^AF488^ signal (blue) in the absence or presence of BCDX2. Each line represents 1 min timepoint. Nucleation rate was calculated for each time frame of the smoothed kymograph by detecting peaks in the AF488 intensity profile. **e,** Kymographs of RAD51 filament growth (by movement of ssDNA into protein + ATP channel) and subsequent disassembly (movement into buffer only channel containing no ATP). Growth and disassembly rates were measured as a slope of the border of the RAD51^AF488^ signal. **f**, Scatter plot (median and IQR) of RAD51 disassembly rates in the absence (n=47 filaments) and presence (n=50 filaments) of BCDX2. Two-sided Mann-Whitney test. **g**, Force measured between the traps as a function of time absence (n=6) or presence of BCDX2 (n=6), B^E144A^CDX2 (n=6), BC^E161A^CDX2 (n=7) and B^E144A^C^E161A^CDX2 (n=6). n values are independent experiments. Shaded area represents SEM.

**Fig. 5:**
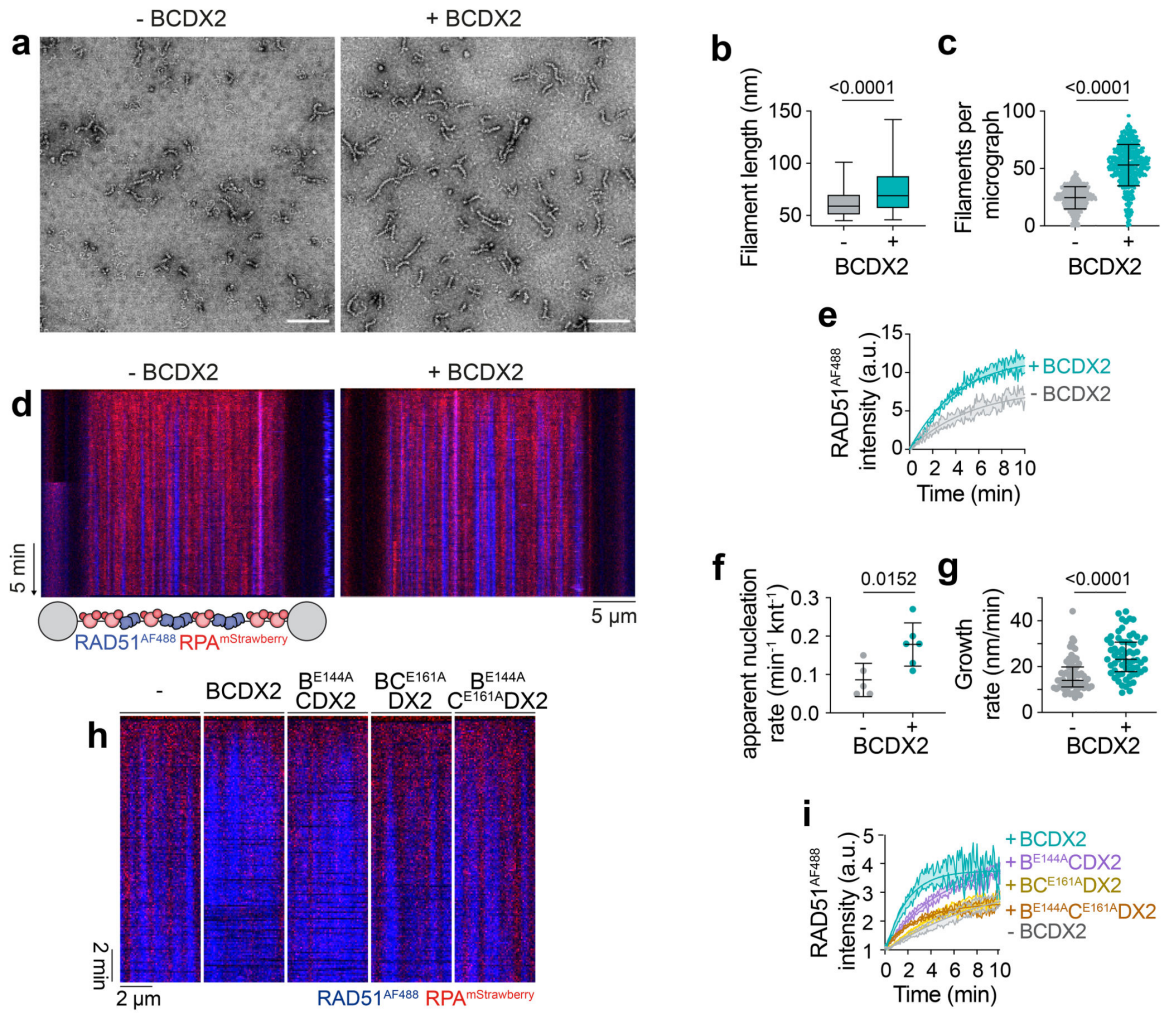
ATP hydrolysis by BCDX2 is required for RAD51 filament growth. **a**, Representative NS-EM micrographs of RAD51 filaments formed in the absence or presence of BCDX2. Scale bar = 100 nm. **b**, Box plot (median, IQR, whiskers = 5 and 95 percentile) of RAD51 filament lengths formed in the absence (n=15,537 filaments) or presence (n=33,436 filaments) of BCDX2. Two-sided Mann-Whitney statistical test. **c**, Scatter plot (mean + s.d.) of RAD51 filaments per micrographs (n=631 micrographs) in the absence or presence of BCDX2. Unpaired two-tailed t- test. **d**, Kymographs showing the displacement of RPA^mStrawberry^ by RAD51^AF488^ in the absence or presence of BCDX2. A schematic of the C-trap experimental set up with fluorescently labelled proteins is shown. **e**, Normalized fluorescence intensity for RAD51^AF488^ signal over time in the absence (n=5) and presence (n=6) of BCDX2. Shaded area represents SEM. Line represents exponential fit. n values represent independent experiments. **f**, Scatter plot (mean and s.d.) of apparent nucleation rates in the absence (n=5) or presence (n=6) of BCDX2. n values represent independent experiments. Unpaired two-tailed t-test. **g**, Scatter plot (median and IQR) of RAD51 filament growth and disassembly rates in the absence (n=65 filaments) and presence (n=64 filaments) of BCDX2. Two-sided Mann-Whitney test. **h**, Kymographs showing the displacement of RPA^mStrawberry^ by RAD51^AF488^ in the absence or presence of BCDX2, B^E144A^CDX2, BC^E161A^CDX2 and B^E144A^C^E161A^CDX2. **i**, Normalized fluorescence intensity for RAD51^AF488^ signal over time in the absence (n=6) or presence of BCDX2 (n=6), B^E144A^CDX2 (n=6), BC^E161A^CDX2 (n=7) and B^E144A^C^E161A^CDX2 (n=6). Shaded areas represent SEM. Lines represent exponential fits. n values represent independent experiments.

We next investigated whether BCDX2 influences RAD51 filament formation. By directly visualising filaments using NS-EM, we found that BCDX2 increases both the quantity (nucleation) and length (growth) of RAD51 filaments (Fig. 5a-c, Extended Data Fig. 6a, b). Next, to monitor RAD51 filament dynamics in real-time, we used a Lumick’s C-trap^39,47^. RAD51 filament assembly was initiated by moving RPA^mStrawberry^-coated λ ssDNA to a protein channel containing Alexa Fluor (AF) 488 labelled RAD51 (RAD51^AF488^) in the absence or presence of BCDX2 (Fig. 5d). Filament formation was monitored by: (i) increased total AF488 fluorescence and (ii) the global rate of assembly with a simultaneous decrease in the force exerted on ssDNA that accompanies filament formation^48^ (Fig. 5e, Extended Data Fig. 6c). The nucleation and growth rates of RAD51 filaments increased 2-fold in the presence of BCDX2, as measured by the frequency of RAD51^AF488^ binding events and the rate of spreading of the fluorescence signal (Fig. 5f, g, Extended Data Fig. 6d). When filaments grown with or without BCDX2 were moved into buffer lacking ATP, they disassembled bidirectionally at the same rate (Extended Data Fig. 6e, f). ATPase deficient BC^E161A^DX2 and B^E144A^C^E161A^DX2 complexes failed to promote RAD51^AF488^ assembly, whereas B^E144A^CDX2 retained partial stimulation relative to wild-type (Fig. 5h, i, Extended Data Fig. 6g). Together, these results show that BCDX2 promotes RAD51 filament nucleation and growth in an ATP hydrolysis dependent manner.

## BCDX2 interacts with ssDNA and RAD51

**Extended Data Fig. 7:**
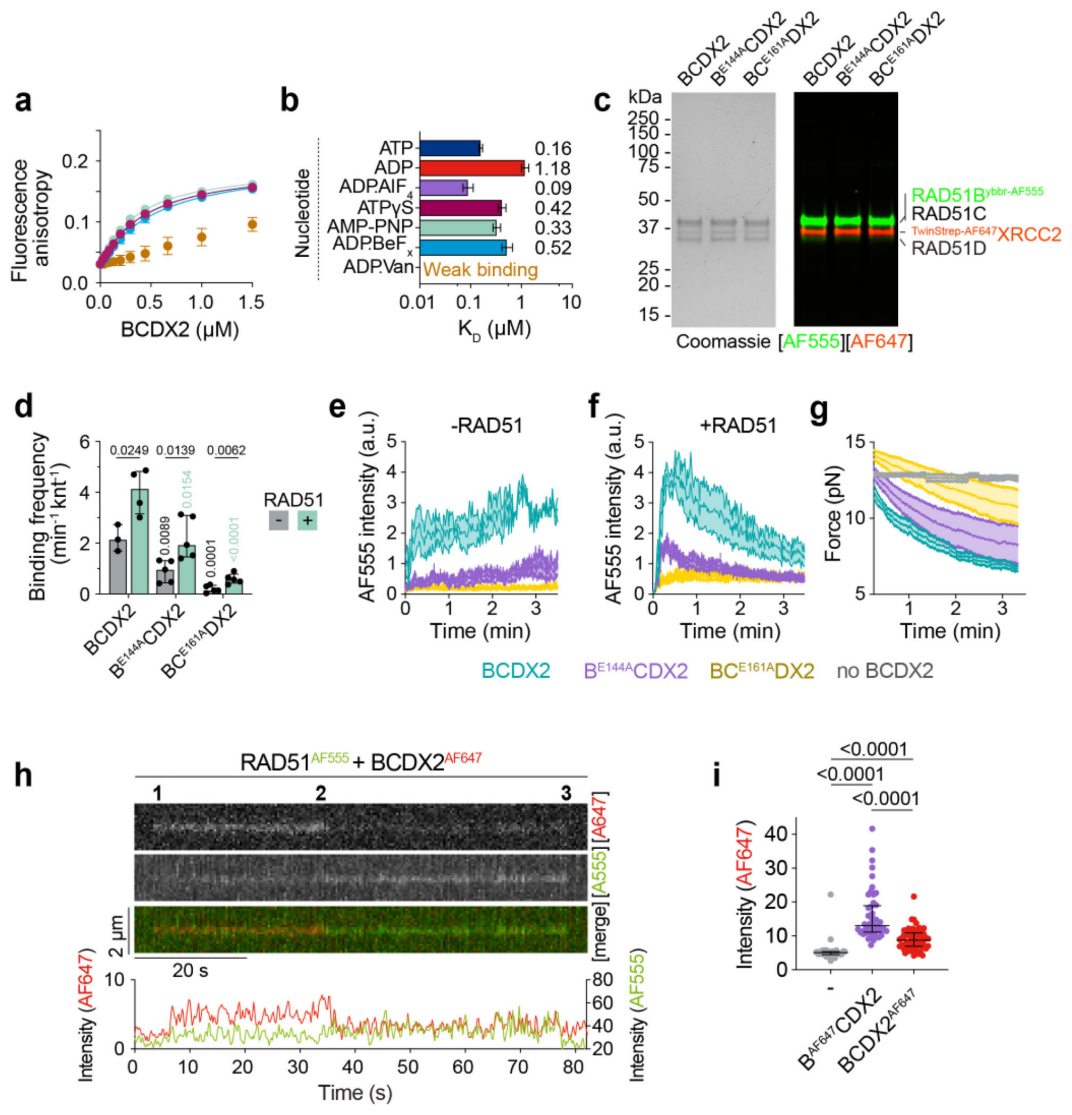
ATP hydrolysis is required for ssDNA binding. **a**, Changes in fluorescence anisotropy upon binding of WT BCDX2 to FAM-dN^15nt^ ssDNA in the presence of ATPyS (n=3, magenta), AMP-PNP (n=3, turquoise), ADP.BeFx (n=3, navy) and ADP.Vanadate (n=3, brown). Lines denote quadratic curve fits. Each point and error bar denotes mean + s.d. n values are independent experiments. **b**, Calculated ssDNA binding affinity constants (centre = *K_D_* values, error bars = 95% CI lower and upper limits) of wild-type BCDX2 in the presence of ATP, ADP, ADP.AlFx, ATPγS, AMP-PNP, ADP.BeFx and ADP.Vanadate. X axis = log_10_ scale. **c**, SDS-PAGE of dual labelled BCDX2, B^E144A^CDX2 and BC^E161A^DX2. Left = Coomassie stain. Right = AF555 and AF647 fluorescence. For gel source data, see Supplementary Figure 1. **d**, Bar chart (mean + s.d.) of binding frequencies of BCDX2, B^E144A^CDX2 and BC^E161A^DX2 to ssDNA in the absence (n=3, n=5, n=5 independent experiments, respectively) or presence (n=4, n=5, n=5 independent experiments, respectively) of RAD51 in the first 30 second window. Unpaired two-tailed t-test. **e** and **f**, Normalized fluorescence intensity for AF555 signal for fluorescently labelled BCDX2, B^E144A^CDX2 or BC^E161A^DX2 over time in the absence (n=3, n=4, n=4 independent experiments, respectively) or presence (n=4, n=5, n=5 independent experiments, respectively) of unlabelled RAD51. Shaded area represents SEM. **g,** Force measured between the traps as a function of time of RAD51 in the absence (n=5) and presence of fluorescently labelled BCDX2 (n=7), B^E144A^CDX2 (n=6) or BC^E161A^DX2 (n=5). n values are independent experiments. Shaded area represents SEM. **h**, Kymographs of RAD51^AF555^/BCDX2^AF647^ FRET. 1 = FRET between RAD51 and BCDX2. 2 = BCDX2 dissociates or RAD51 binding. 3 = RAD51 dissociates. **i**, Scatter plot of AF647 intensity values for RAD51 alone (bleed-through) (n=39), B^AF647^CDX2 (n=48) and BCDX2^ybbr-AF647^ (n=61). Median + IQR. Two-sided Mann-Whitney statistical test.

**Fig. 6:**
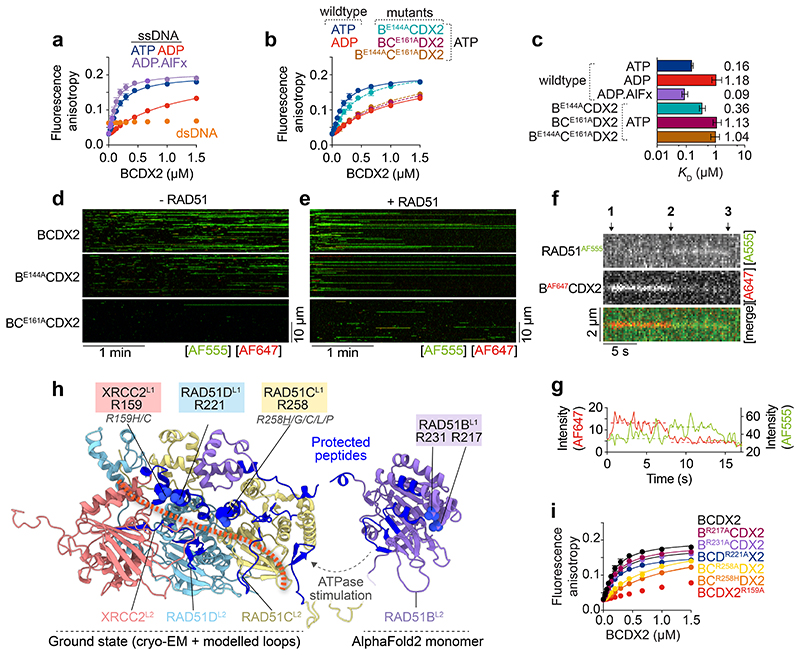
Interaction of BCDX2 with ssDNA and RAD51. **a,** Changes in fluorescence anisotropy upon binding of wild-type BCDX2 to FAM-dN^15nt^ ssDNA in the presence of ATP (n=17, blue), ADP.AlFx (n=3, purple) or ADP (n=12, red), and to FAM-dN^30bp^ dsDNA (n=3, orange). Lines denote quadratic curve fits. Each point and error bar denotes mean + s.d. **b**, As **a,** but including binding of B^E144A^CDX2 (n=3, turquoise), BC^E161A^DX2 (n=3, magenta) and B^E144A^C^E161A^DX2 (n=3, brown) to FAM-dN^15nt^ in the presence of ATP. n values are independent experiments. **c**, Calculated ssDNA binding affinity constants from curves in panels **a** and **b** (centre = *K*_D_ values, error bars = 95% CI lower and upper limits). X axis = log_10_ scale. **d** and **e**, Kymographs showing binding of dual-labelled AF555/AF647 BCDX2, B^E144A^CDX2, and BC^E161A^DX2 to λ ssDNA in the absence or presence of unlabelled RAD51. **f**, Kymographs showing FRET between RAD51^AF555^ and B^AF647^CDX2. 1 = FRET between RAD51 and BCDX2. 2 = BCDX2 dissociates. 3 = RAD51 dissociates. **g**, Fluorescence intensity profile showing anti-correlated emission for AF555 and AF647. **h,** Comparative HDX-MS analysis of BCDX2-ADP.AlFx-ssDNA vs BCDX2- ADP.AlFx. Regions protected from deuterium uptake after 300 secs are indicated (blue). The L1 and L2 loops of RAD51B, RAD51C, RAD51D and XRCC2 and putative arginine residues that engage ssDNA are shown. Italicised are VUS, underlined are confirmed pathogenic mutations. Dashed orange line represents the putative path of ssDNA. **i**, Changes in fluorescence anisotropy upon binding of BCDX2, or the indicated mutants, to FAM-dN^15nt^ ssDNA in the presence of ATP (all n=3, except wt n=17, n values are independent experiments). Results for wt originally shown in Fig 6a. Lines denote quadratic curve fits. Each point and error bar denotes mean + s.d.

In contrast to RAD51, which binds both ssDNA and dsDNA^17^, BCDX2 binds specifically to ssDNA, as determined by fluorescence anisotropy (Fig. 6a). The ssDNA binding affinity in the presence of ATP (0.16 μM) was ~7-fold greater than in the presence of ADP (1.18 μM), with the highest affinity measured in the presence of the transition state mimetic ADP.AlFx (0.09 μM) showing that ATP hydrolysis promotes high-affinity ssDNA binding. Binding affinities ranging from 0.33 - 0.52 μM were observed for the pre-hydrolysis ATP mimetics AMP-PNP, ATPyS, and ADP.BeFx. The presence of ADP.Vanadate, which traps ADP in the nucleotide binding site by mimicking the transition state of the γ-phosphate of ATP during ATP hydrolysis, reduced ssDNA binding (Extended Data Fig. 7a, b).

In the presence of ATP, the ssDNA-binding affinities of the BC^E161A^DX2 and B^E144A^C^E161A^DX2 ATPase-deficient mutants were similar to the affinity of wild-type protein with ADP (Fig. 6b), showing that they fail to induce the high affinity DNA binding state. However, inactivation of RAD51B’s ATPase (B^E144A^CDX2), resulted in only a small decrease in affinity consistent with its partial stimulation of RAD51 filament assembly (Fig. 6b, c). These observations were confirmed by monitoring λ ssDNA binding by fluorescently labelled BCDX2, B^E144A^CDX2 and BC^E161A^DX2 (Fig. 6d, Extended Data Fig. 7c, d). These results, together with the comparative HDX-MS experiments of ADP.AlFx/ADP and ADP.BeFx/ADP (Fig. 4h, Extended Data Fig. 5i), show that high-affinity ssDNA binding is driven through RAD51B^CTD^-RAD51C^CTD^ interactions that occur during ATP hydrolysis.

The frequency and stability of interactions between BCDX2 and ssDNA increase in the presence of RAD51 (Fig. 6e, Extended Data Fig. 7d). This effect was temporal, as it occurred only at early time points with BCDX2 dissociating as the RAD51 filaments assembled (detected through force decrease with time; Extended Data Fig. 7e-g). To provide direct evidence for BCDX2 and RAD51 interaction, we monitored Förster Resonance Energy Transfer (FRET) between RAD51^AF555^ and B^AF647^CDX2 on λ ssDNA (Fig. 6f, g). Three distinct events were observed: (i) initial B^AF647^CDX2 fluorescence emission (FRET-mediated via interaction of B^AF647^CDX2 with RAD51^AF555^), (ii) RAD51^AF555^ fluorescence (with loss of B^AF647^CDX2 signal upon dissociation), and finally (iii) a loss of fluorescence emission as RAD51^AF555^ dissociates/bleaches. These results indicate that BCDX2 and RAD51 interact and associate with ssDNA, followed by subsequent BCDX2 dissociation. FRET experiments were also carried out with BCDX2^AF647^ and similar results were observed (Extended Data Fig. 7h) although the fluorescence intensity of B^AF647^CDX2 was greater than BCDX2^AF647^ following FRET with RAD51^AF555^ (Extended Data Fig. 7i). These results indicate that RAD51 interacts with BCDX2 via RAD51B.

**Extended Data Fig. 8:**
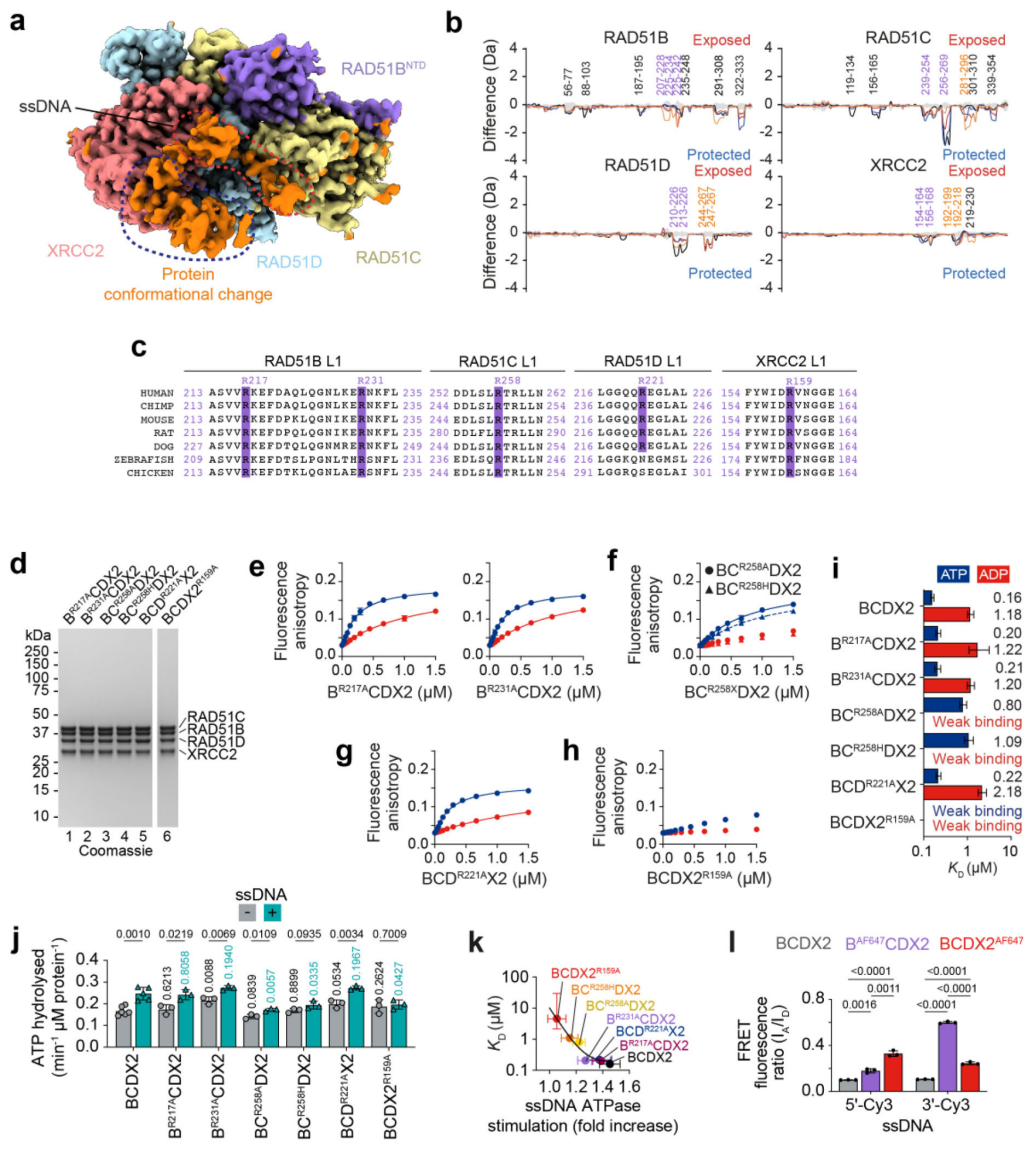
Mechanism of ssDNA binding by BCDX2 **a**, Cryo-EM model of BCDX2 bound to ssDNA. Unmodelled density of ssDNA is labelled (red) and an additional density, thought to be due to a protein conformation change, is indicated (blue). **b,** Difference plot between BCDX2-ADP.AlFx and BCDX2-ADP.AlFx-ssDNA showing level of deuterium uptake after 3 (orange), 30 (red), 300 (blue) and 3000 (black) seconds. Positive values = exposure (red); negative values = protection (blue). Purple text = L1 loops. Orange text = L2 loops. RAD51B: 110 peptides, 97.7% coverage, 3.38 redundancy. RAD51C: 113 peptides, 98.1% coverage, 3.32 redundancy. RAD51D: 108 peptides, 98.5% coverage, 3.88 redundancy. XRCC2: 89 peptides, 97.9% coverage, 3.27 redundancy. **c,** Conservation of putative ssDNA binding arginine residues in the L1 loops of RAD51B, RAD51C, RAD51D and XRCC2. **d**, SDS-PAGE of mutant BCDX2 mutant proteins. For gel source data, see Supplementary Figure 1. Fluorescence anisotropy ssDNA binding curves in the absence of ATP (blue) or ADP (red) for **e**, B^R217A^CDX2 (n=3) and B^R231A^CDX2 (n=3), **f**, BC^R258A^DX2 (n=3) and BC^R258H^DX2 (n=3), **g**, BCD^R221A^X2 (n=3), and **h**, BCDX2^R159A^ (n=3). n values are independent experiments. Lines denote quadratic curve fits. Each point and error bar denotes mean + s.d. **i,** Calculated ssDNA binding affinity constants (centre = *K_D_* values, error bars = 95% CI lower and upper limits) of BCDX2 arginine mutants. X axis = log_10_ scale. **j**, Bar chart (mean + s.d.) of ATP hydrolysis rate. Unpaired two-tailed t-test. All n=3, except wt which is n=6. n values represent independent experiments. **k,** Correlation of ssDNA binding affinity (errors = 95% CI) against fold increase in ssDNA ATPase stimulation (mean + s.d.). Curve fitting by exponential decay curve. **l**, Bar chart (mean + s.d.) of FRET fluorescence ratio (I_A_/I_D_) between 5′-Cy3-dN^15nt^ or dN^15nt^-Cy3-3′ and BCDX2, B^AF647^CDX2 and BCDX2^AF647^. All n=3 independent experiments. Two tailed unpaired t-test.

Initial attempts to determine the structure of the BCDX2-ssDNA complex by cryo-EM were unsuccessful due to low occupancy of bound ssDNA. We therefore performed partial signal subtraction and focused classification without alignment on the region containing the L1 DNA binding loops of RAD51C and RAD51D, plus the L2 DNA binding loops of RAD51C, RAD51D and XRCC2. We obtained a 2.9 Å cryo-EM map of BCDX2 bound to ssDNA (Extended Data Figs 1 and 3). In addition to the ssDNA, we observed an additional uncharacterised density due to a conformation change in XRCC2 (Extended Data Figure 8a). Unfortunately, the additional density was not of sufficient quality or resolution to enable atomic model building. The presence of ssDNA, however, was confirmed using HDX-MS, by comparisons of BCDX2 with and without ssDNA. We observed protection from deuterium uptake for the L1 DNA binding loops of RAD51C, RAD51D and XRCC2, as well as the L2 DNA binding loops of RAD51C and RAD51D (Fig. 6h, Extended Data Fig. 8b).

Alignment of the RAD51B, RAD51C, RAD51D and XRCC2 sequences, in human and model organisms (Extended Data Fig. 8c), identified key arginine residues in the L1 loops that could interact with the phosphate backbone of ssDNA analogous to RAD51^R229/R241^. We therefore purified BCDX2 complexes containing mutations at these sites and measured ssDNA binding (Fig. 6i, Extended Data Fig 8d-i). In agreement with HDX-MS data, which showed little protection from deuterium uptake for the RAD51B L1 loop in the presence of ssDNA, the affinities of B^R217A^CDX2 and B^R231A^CDX2 towards ssDNA were similar to wild-type protein. However, mutation of RAD51C^R258A^ in the L1 loop, which is rotated upon ATPase activation, resulted in a severe defect in ssDNA binding. Similar results were obtained with the RAD51C^R258H^ mutation, providing molecular insights into Fanconi anaemia pathogenesis for individuals carrying this mutation^7,12^. Mutation of RAD51D^R221^ had minimal effects, whereas ssDNA binding was mostly ablated by mutation of XRCC2^R159^, another residue associated with VUS (Supplementary Table 1). These results show that ssDNA binding by BCDX2 is mostly driven by interaction with RAD51C and XRCC2, but not RAD51B or RAD51D. In the absence of ssDNA, all arginine mutants exhibited similar ATPase activity as the wild-type protein (Extended Data Fig. 8j). However, in the presence of ssDNA, ATPase stimulation was directly correlated with ssDNA binding affinity (Extended Data Fig. 8k), driven through increased RAD51B^CTD^ and RAD51C^CTD^ interaction as determined by HDX-MS (Fig. 6h).

To determine the orientation of ssDNA binding, FRET was monitored between 5′- or 3′-Cy3 labelled ssDNA and BCDX2 labelled with RAD51B^AF647^ or XRCC2^AF647^. The FRET ratios were highest between 5′-Cy3 labelled ssDNA and BCDX2^AF647^, and also between 3′-Cy3 labelled ssDNA and B^AF647^CDX2, indicating that the orientation of BCDX2 binding relative to ssDNA is 3′ to 5′ (Extended Data Fig. 8l). As RAD51B interacts directly with RAD51, these results indicate that the nucleoprotein filament grows preferentially with a 3′ to 5′ polarity.

**Extended Data Fig. 9:**
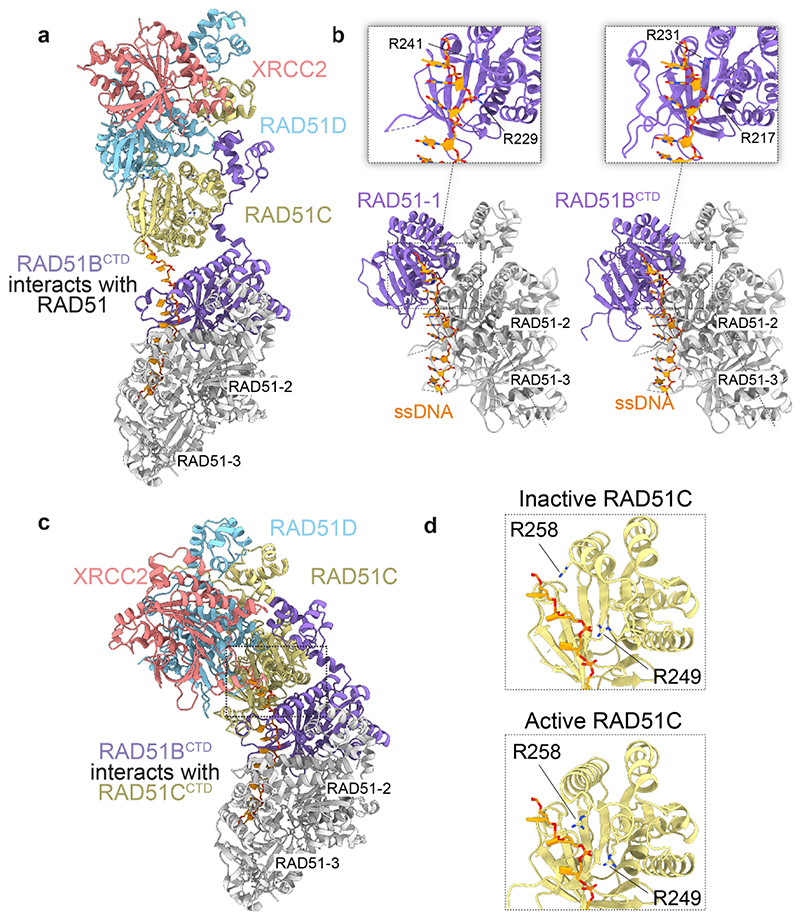
Interplay between BCDX2 and RAD51 filaments. **a,** RAD51 B binds to ssDNA and RAD51. **b**, Structure of the RAD51 filament (PDB = 5H1B) bound to ssDNA (left). Modelling of RAD51B^CTD^ on RAD51-1 (rmsd = 1.024 Å) (right). Zoomed view (upper panels) showing engagement of RAD51^R229/R241^ and RAD51B^R217/R231^ with ssDNA. **c**, RAD51B interacts RAD51C during ATP hydrolysis, promoting high affinity ssDNA binding by the BCDX2 complex. **d**, Structural modelling of RAD51C binding a triplet of nucleotides in both RAD51C ground and active intermediate conformations.

In summary, we have defined the structure and dynamic properties of the BCDX2 complex and provided new insights into its relationships and interactions with RAD51. We find that BCDX2 promotes RAD51 filament nucleation and extension, in events dependent upon BCDX2′s high-affinity ssDNA binding state induced by the coupled RAD51B and RAD51C ATPases. Previous studies indicated that RAD51 paralog complexes in lower eukaryotes promote filament growth by the transient capping of filament ends^38–40^. Our data with human BCDX2 are consistent with such a model, with RAD51B providing a bridge between the CDX2 structural core and RAD51 (Extended Data Fig. 9a, b). The AlphaFold2 model of RAD51B^CTD^ perfectly mimics a RAD51^CTD^ protomer and indicates that the CTD maintains contacts with the phosphodiester backbone of ssDNA via RAD51B^R217/R231^, analogous to RAD51^R229/R241^. We suggest that the flexible RAD51B^ctd^ binds to RAD51, and that this engagement provides initial filament stabilisation (nucleation). Next, ATP hydrolysis by RAD51C induces an interaction with RAD51B^CTD^, resulting in high affinity ssDNA binding by the CDX2 core (Extended Data Fig. 9c). By modelling nucleotide triplet binding by RAD51C, we found that RAD51C^R241/R258^ occupies the same geometry as RAD51^R229/R241^, in the active intermediate but not ground state (Extended Data Fig. 9d), validating the necessity of RAD51C^R258^ rotation for high affinity ssDNA binding.

The ATP-bound subunits RAD51D-XRCC2 would cap and stabilise the filament through XRCC2^R159^ interacting with ssDNA, whereas the absence of an NTD in XRCC2 would prevent BCDX2 from intercalating into RAD51 filaments. Through repeated rounds of ATP hydrolysis, BCDX2 would cycle through high (binding) and low (dissociation) affinity ssDNA binding states, which may in turn allow repeated cycles of filament stabilisation. One possibility is that BCDX2 is capable of limited translocation along the ssDNA, such that filament extension could occur through a ratchet-like mechanism involving RAD51B^CTD^-RAD51 interactions. Net RAD51 filament growth would then occur with a 3′-5′ polarity consistent with the directionality of RAD51-mediated strand exchange^49^. This mechanism explains the critical contributions of the RAD51 paralogs to DNA repair, and how dysfunction in their ATPase or ssDNA-binding activities may contribute to genomic instability.

## Methods

### Expression vectors

The RAD51 expression vector, pCH1-RAD51, was derived from pCH1-RAD51opt^50^ by removal of the GroES and GroEL sequences. *RAD51B, RAD51C, RAD51D* and *XRCC2* were individually cloned into pFastBac1 modified with an expression enhancer and Kozak sequence upstream of the coding sequence^51^. An N-terminal TwinStrep-TEV sequence was included for *XRCC2* (*TwinStrep-TEVXRCC2)*. All four genes were assembled into the same plasmid with Gibson assembly to generate pBIG-BCDX2^52^. The construct was validated by restriction enzyme digestion with SwaI or PstI, and nanopore sequencing (Plasmidsaurus). Point, truncating and labelling mutants *RAD51B^ntd^* (deletion of amino acids 64-350), *RAD51B^E144A^, RAD51B^R217A^, RAD51B^R231A^, RAD51B^K324A^, RAD51B^ybbr^* (with a sequence to encode a C-terminal DSLEFIASKLA ybbr motif ^53^ and preceding GGGSGGG linker), *RAD51B^ybbr/E144A^, RAD51C^E161A^, RAD51C^R258A^, RAD51C^R258H^, RAD51D^R221A^, XRCC2^R159A^* and *XRCC2^K261A^* were generated by around-the-horn-mutagenesis of the parent pFastBac vectors. They were then assembled into pBIG to generate pBIG-B^NTD^CDX2, pBIG-B^E144A^CDX2, pBIG-B^R217A^CDX2, pBIG-B^R231A^CDX2, pBIG-B^ybbr/E144A^CDX2, pBIG-B^K324A^CDX2, pBIG-BC^E161A^DX2, pBIG-BC^R258A^DX2, pBIG- BC^R258H^DX2, pBIG-BCD^R221A^X2, pBIG-BCDX2^R159A^, pBIG-BCDX2^K261A^, pBIG- B^E144A^C^E161A^DX2, and pBIG-B^ybbr^C^E161A^DX2.

### Protein purification

For expression of proteins in insect cells, bacmids, primary and secondary baculoviruses were generated following protocols outlined for the Bac-to-bac system (Life technologies). Baculovirus titres were quantified by RT-PCR^54^. For the expression of BCDX2, and all point, truncating and labelling mutants, secondary baculoviruses were used to infect (viral multiplicity of infection [MOI] = 0.5) Sf9 insect cells (0.5 L) grown in Sf-900 III serum free media (Gibco) seeded at a density of 2 x 10^6^ cells/mL. Cells were grown at 27°C with continuous agitation (140 rpm) for 66 hours. Sf9 cells were sourced from the Structural Biology Science Technology Platform, The Francis Crick Institute.

All purifications were carried out at 4°C. Cells were collected by centrifugation (1,500 g, 10 min), washed once with phosphate buffered saline (PBS; 137 mM NaCl, 2.7 mM KCl, 10 mM Na_2_HPO_4_, 1.8 mM KH_2_PO_4_), and re-pelleted (1,500 g, 10 min). The cell pellet was resuspended in 75 mL of lysis buffer (25 mM HEPES pH 7.5, 10% (v/v) glycerol, 2.5 mM MgCl_2_, 0.25 mM TCEP, 500 mM NaCl, 2.5 mM ATP, 0.05% Triton X-100 and HALT protease and phosphatase inhibitors) and sonicated using the QSonica Q700 (25 mA, 2 s on/off, 5 min processing time). Insoluble matter was separated by centrifugation (Beckmann J-26 centrifuge and JA25.5 rotor, 60,000 g, 45 minutes). The clarified lysate was incubated with 2.5 mL StrepTactin XT 4-flow resin (IBA Lifesciences) for 1 hour with rotation. The resin was pelleted (500 g, 5 min) and transferred to an Econo-Pac gravity flow column (BioRad). The resin was washed with 5 CV (column volumes) of BCDX2 lysis buffer, 5 CV of HGMT buffer (25 mM HEPES pH 7.5, 10% (v/v) glycerol, 2.5 mM MgCl2 and 0.25 mM TCEP) containing 300 mM NaCl and 1 mM ATP and 10 CV of HGMT buffer containing 150 mM NaCl and 0.5 mM ATP. The resin was resuspended in 1 CV purification buffer containing 150 mM NaCl, 0.5 mM ATP and 50 mM biotin and mixed on a roller for 30 minutes. The eluate was collected, and a further 4 CV eluted by gravity flow. The eluted protein concentration was measured using protein assay dye reagent (BioRad) and mixed with a 1:20 (c/c) molar ratio of TEV protease to remove the TwinStrep tag. Following overnight cleavage, the protein was passed through a 0.22 μm filter and loaded onto a 1 mL Resource Q column (Cytiva) equilibrated in HGMT buffer containing 150 mM NaCl using an ÄKTA Pure HPLC purification system (Cytiva) controlled by UNICORN software. Bound protein was washed with 3 CV of HGMT buffer containing 150 mM NaCl and eluted from the column using an 8 CV gradient with HGMT buffer containing 1 M NaCl. Fractions containing BCDX2 were pooled and loaded onto a Superdex 200 Increase 10/300 column (Cytiva) equilibrated in HGMT buffer containing 150 mM NaCl on an ÄKTA pure. Peak fractions were pooled, flash-frozen in liquid nitrogen and stored at -80°C. B^ybbr^CDX2 was purified as described for BCDX2, with TEV cleavage and gel filtration omitted.

pCH1-RAD51 was used for the expression of RAD51. BL21 Star (DE3) (Life Technologies) were initially transformed with pGro7 (GroEL-GroES) and transformants selected on LB plates containing 20 μg/mL chloramphenicol to generate GroEL-ES competent cells (TaKaRa). These were subsequently transformed with pCH1-RAD51 and transformants were selected using 50 μg/mL kanamycin. A starter culture was grown overnight (37°C, 220 rpm) in Luria Broth (LB) containing 50 μg/mL kanamycin and 0.8% glucose (v/v), and then expanded to 2 L of culture with a starting optical density (OD) at 600 nm of 0.1 the following day (37°C, 200 rpm). Expression of GroEL-GroES was induced at OD_600nm_ = 0.5 by the addition of 0.5 mg/mL L-arabinose and the incubator was cooled to 18°C. After 30 min, the expression of RAD51 was induced by the addition of 0.5 mM isopropyl β-d-1-thiogalactopyranoside (IPTG) and the culture was grown overnight. Cells were collected by centrifugation (1,500 g, 10 min), washed once with PBS and re-pelleted (1,500 g, 10 min). The cell pellet was resuspended in 40 mL RAD51 lysis buffer (50 mM HEPES pH 8, 10% (v/v) glycerol, 2 mM EDTA, HALT protease inhibitors and 0.25 mM TCEP) and passed through an Emusiflex-C5 (Avestin) 3x with applied pressure between 15,000 - 20,000 psi to lyse the bacteria. Triton X-100 (0.05%) was added, and the lysate was sonicated using a QSonica Q700 (25 mA, 2 s on/off, 5 min processing time). Insoluble matter was separated by centrifugation (Beckmann J-26 centrifuge and JA25.5 rotor, 60,000 g, 45 minutes). Spermidine was then added dropwise from a stock solution to the clarified lysate, to achieve a final concentration of 7 mM. The precipitate was collected by centrifugation (15,000 g, 10 min), and resuspended in RAD51 resuspension buffer (25 mM HEPES pH 8, 10% (v/v) glycerol, 0.25 mM TCEP) containing 150 mM NaCl. This precipitate was again collected by centrifugation, and the process repeated four more times with RAD51 resuspension buffer containing 250, 300, 500 and 600 mM NaCl. Fractions containing RAD51 were pooled and loaded directly onto three 5 mL HiTrap Heparin HP columns (Cytvia) attached in tandem, which were equilibrated in RAD51 purification buffer (25 mM HEPES pH 7.5, 5% (v/v) glycerol, 1 mM EDTA, 0.25 mM TCEP) containing 300 mM NaCl. RAD51 was eluted with a 10 CV gradient using RAD51 purification buffer containing 2 M NaCl. The RAD51 was pooled, and the salt concentration was decreased by dropwise addition of RAD51 purification buffer to a final concentration of 80 mM NaCl. RAD51 was then applied to a 5 mL HiTrap SP HP (Cytiva) column equilibrated in RAD51 purification buffer containing 80 mM NaCl, and the flow through was subsequently loaded onto two HiTrap Q HP (Cytiva) columns attached in tandem, which were equilibrated in RAD51 purification buffer containing 80 mM NaCl. RAD51 was eluted with a 10 CV gradient using RAD51 purification buffer containing 1 M NaCl. Fractions containing RAD51 were pooled, and the majority was flash frozen in 10 mg aliquots, stored at -80°C and reserved as a master stock for later polishing. For the working stock, a 10 mg aliquot of RAD51 was thawed and diluted to 100 mM NaCl with the addition of RAD51 purification buffer. RAD51 was loaded onto a 1 mL MonoQ 5/50 GL column (Cytiva) equilibrated in RAD51 purification buffer with 100 mM NaCl, and subsequently eluted with 8 CV of RAD51 purification buffer containing 1 M NaCl. Fractions were pooled and dialysed overnight into RAD51 storage buffer (25 mM HEPES pH 7.5, 10% glycerol (v/v), 150 mM NaCl, 0.5 mM EDTA, 0.25 mM TCEP). 5 μL aliquots of RAD51 were flash frozen in liquid nitrogen and stored at -80°C. RAD51 was free of contaminating nuclease activity and exhibited the expected activity in DNA strand exchange reactions.

RAD51^C319S^ used for fluorescent labelling and single-molecule experiments was purified as described^47^. RPA^mStrawberry^ was purified as described for RPA^eGFP 47^

### Fluorescent labelling of proteins

RAD51^C319S^ was fluorescently labelled with AF488 or AF555 as described^47^. BCDX2 was single and dual (FRET) labelled using the ybbr/Sfp transferase and LPXTG/sortase labelling strategies^53,55^. For XRCC2 LPXTG/sortase labelling, Resource Q purified B^ybbr^CDX2^TS^ was mixed with a 10-fold molar excess of AF647 labelled peptide (NH2-C^AF647^HHHHHHHHHHLPETGG-COOH), recombinant sortase enzyme and 5 mM MgCl_2_, and incubated at 4°C overnight to yield B^ybbr^CDX2^AF647^. For RAD51B ybbr/Sfp transferase labelling, B^ybbr^CDX2^TS^ was mixed with a 3-fold molar excess of CoA-AF647, Sfp transferase enzyme, 5 mM MgCl_2_, and incubated overnight at 4°C to yield B^AF647^CDX2. For dual labelling, B^ybbr^CDX2^TS^, B^ybbr/E144A^CDX2^TS^ or B^ybbr^C^E161A^DX2^TS^ were mixed with a 10-fold molar excess of AF647 labelled peptide, a 3-fold molar excess of CoA-AF555, Sfp and sortase enzymes, 5 mM MgCl_2_ and incubated at 4°C overnight to yield B^AF555^CDX2^AF647^, B^AF555/E144A^cdX2^AF647^ and b^AF555^C^E161A^DX2^AF647^, respectively. All fluorescently labelled proteins were gel filtered on a Superdex 200 Increase (either 10/300 or 3.2/100 GL) column (Cytiva) on an ÄKTA Pure to separate protein from fluorescent peptides/molecules and labelling enzymes.

### Limited proteolysis

BCDX2 (15 μM) was incubated for 4 hours at 37°C with and without chymotrypsin (1.5 μM) in HGMT buffer containing 150 mM NaCl, and 0.5 mM ADP.BeF_x_ (0.5 mM ADP, 0.5 mM BeSO_4_ and 10 mM NaF). The samples were then gel filtered using a Superdex 200 Increase 3.2/300 GL column (Cytiva) equilibrated in HMT buffer (25 mM HEPES pH 7.5, 2.5 mM MgCl_2_, 0.25 mM TCEP) with 100 mM NaCl on a Micro-kit equipped ÄKTA pure. Fractions were taken and analysed on 5 SDS-PAGE gels. One was directly visualised with Quick Coomassie stain (Generon) and visualised on a ChemiDoc MP Imaging system. The others were analysed by western blotting with antibodies against RAD51B (Rabbit polyclonal, 1:1000 SWE32, this lab), RAD51C (Rabbit polyclonal, 1:1000, SWE68^56^), RAD51D (Rabbit monoclonal, 1:1000, Abcam ab202063) and XRCC2 (Rabbit polyclonal, 1:1000, SWE35^56^). Membranes were incubated with Alexa Fluor Plus 800 anti-rabbit secondary antibody (1:40,000, Invitrogen A32735) and imaged using an Odyssey DLx instrument with ImageStudio software (Licor).

### Oligonucleotides

All DNA oligonucleotides were HPLC purified (Integrated DNA Technologies). The names and sequences of the oligos are as follows, whereby FAM is 6-carboxyfluoroscein: FAM-dN^12nt^ (5′-FAM-TATCGAATCCGT-3′), FAM-dN^15nt^ (5′-FAM-TATCGAATCCGTCTA-3′), FAM-dN^30nt^ (5′-FAM-TATCGAATCCGTCTAGTCAACGCTGCCGAA-3’), 5′-Cy3-dN^15nt^ (5′-Cy3-TATCGAATCCGTCTA-3′), dN^15nt^-Cy3-3′ (5’-TATCGAATCCGTCTA-Cy3-3′), dN^12nt^ (5′-TATCGAATCCGT-3′), dN^15nt^ (5′-TATCGAATCCGTCTA-3′), dN^30nt^ (5′-TATCGAATCCGTCTAGTCAACGCTGCCGAA-3′) and dN^rc30nt^ (5′-FAM-TTCGGCAGCGTTGACTAGACGGATTCGATA-3′). To generate 6FAM-dN^30bp^ dsDNA, equimolar concentrations of FAM-dN^30nt^ and dN^30nt^-rc were mixed in 10 mM Tris-HCl pH 7.5, 100 mM NaCl and 1 mM EDTA, heated to 90°C and gradually cooled to room temperature. Concentrations were measured using a spectrophotometer using absorbance values at 260 nm. Substrates were stored at -20°C.

### Fluorescence anisotropy

Fluorescence anisotropy was measured as technical duplicates of 10 μL reactions in 384 well low volume microplates (Corning). Proteins were diluted in HGMT buffer containing 150 mM NaCl. Samples (5 μL) were mixed with an equal volume of 100 nM fluorescein labelled oligonucleotides in HMT buffer containing 50 mM NaCl, 0.001% Brij-35 and 2 mM nucleotide (ATP, ADP, ATPγS, AMP-PNP, ADP.BeFx [2 mM ADP, 2 mM AlCl3 20 mM NaF], ADP.AlFx [2 mM, ADP, 2 mM BeSO4, 20 mM NaF] or ADP.Vanadate [2 mM ADP, 2 mM vanadate]), such that the final oligonucleotide, nucleotide and Brij-35 concentration equalled 50 nM, 1 mM and 0.0005% respectively. The mix was incubated for 30 minutes at 25°C, and fluorescence anisotropy measurements were made on a Clariostar plate reader (BMG Labtech). Blank corrected anisotropy measurements were generated using MARS software (Labtech) and averaged across independent experiments, plotted against protein concentration and curve fitted using the following quadratic equation in GraphPad Prism 9 to determine *K_D_* values:

$$Y=A_{\min}+(A_{\max}-A_{\min})\times \frac{x+L+K_{\text{D}}-\sqrt{{\left(x+L+K_{\text{D}}\right)}^{2}-4\times x\times L}}{2\times L}$$

whereby Y = fluorescence anisotropy, A_min_ and A_max_ = minimum and maximum fluorescence anisotropy values, L = ligand concentration (equal to 0.05 μM), x = protein concentration and *K_D_* = dissociation constant.

### Bulk ssDNA-BCDX2 FRET

BCDX2, fluorescently labelled B^AF647^CDX2 or BCDX2^AF647^, was mixed with either dN^15nt^, 5′-Cy3-dN^15nt^ or dN^15nt^-Cy3-3′ ssDNA in 10 μL of 25 mM HEPES pH 7.5, 5% glycerol, 2.5 mM MgCl_2_, 100 mM NaCl, 0.25 mM TCEP, 1 mM ADP.AlFx (1 mM ADP,1 mM AlCl_3_, 10 mM NaF) in 384 well low volume microplates (Corning). Fluorescence emission spectra from 550 nm to 800 nm were measured following excitation at 500 nm on a Clariostar plate reader (BMG Labtech). The ratio of fluorescence intensity (I_A_/I_D_) at 647 nm and 555 nm was measured for all combinations of protein and ssDNA.

### HPLC analysis of bound nucleotides

Resource Q purified wild-type and mutant BCDX2 were incubated for 1 hour at RT in the absence of nucleotide, or in the presence of 1 mM ATP, ADP or ATPγS, and then gel filtered using a Superdex 200 Increase 3.2/300 GL column (Cytiva) equilibrated in HMT buffer containing 100 mM NaCl (or the equivalent buffer lacking 2.5 mM MgCl_2_) on a Micro-kit equipped ÄKTA pure. Protein fractions were pooled, and absorbance spectra were measured on a Jasco V-760 spectrophotometer operated by SpectraManager software. Curves were adjusted to correct for any light scattering, and the protein concentrations were calculated by Beer-Lambert law, using absorbance values at 280 nm. Extinction coefficients (BCDX2 = 80,220 M^-1^ cm^-1^) were adjusted to include 3-4 molecules of nucleotide (ATP/ADP = 2,390 M^-1^ cm^-1^ at 280 nm). Nucleotides were extracted from the protein sample by the addition of 0.7% perchloric acid and 200 mM sodium acetate, incubated (10 min, RT) and centrifuged (15,000 g, 10 min) to clear any insoluble matter. The supernatant was diluted with HPLC buffer (100 mM K_2_HPO_4_/KH_2_PO_4_ pH 6.5, 10 mM tetrabutylammonium bromide) with 12% acetonitrile and passed through a Durapore- PVDF (0.22 μm) centrifugal filter (Millipore, 16,000 g, 2 minutes).

The nucleotide content of BCDX2 was determined by reverse-phase HPLC (RP-HPLC) using ion pair chromatography. Samples (55 μL) were applied to a Zorbax SB-C18 (4.6 × 250 mm, 3.5 μm, 80 Å pore size, Agilent Technologies) column maintained at 30°C and mounted onto a Jasco HPLC system controlled by Chromnav software (v1.19 Jasco). Nucleotides were separated by running the column at 1 mL/min in HPLC buffer containing 12% acetonitrile. Samples containing ATPγS were run isocratically with 12% acetonitrile for 10 mL followed by a gradient from 12% to 20% acetonitrile over 20 mL. Absorbance from the column eluent was continually monitored between 200 and 650 nm (1 nm intervals) using an MD-2010 photodiode array detector (Jasco). Nucleotides were quantified from the peak integrals in the 260 nm absorbance channel and concentrations were calculated using ATP and ADP standard curves. Statistical analyses and figure plotting were performed using GraphPad Prism 9.

### ATPase assay

Wild-type or mutant BCDX2 (1 μM) were mixed with 15 μM cold ATP and 0.1 μCi/μL α-^32^P-ATP, with and without dN^15nt^ ssDNA (3 μM) in 25 mM HEPES pH 7.5, 5% glycerol, 100 mM NaCl, 2.5 mM MgCl_2_ and 0.25 mM TCEP. Samples were incubated (37°C, 30 min) before quenching with 10 mM EDTA. α-^32^P-ATP and α-^32^P-ADP were separated by chromatography on TLC plates (Macherey-Nagel) using 500 mM LiCl and 1 M formic acid buffer. Plates were dried, exposed to a phosphoscreen and imaged on a Typhoon 9500 phosphorimager. Percentage ATP hydrolysis was calculated using Fiji (ImageJ). Statistical analyses and figure plotting were performed using GraphPad Prism 9.

### Single-molecule assays

Experiments were performed using a Lumicks C-trap operated by Bluelake software. The flow cell was washed before each experiment as described^39,47^. Biotinylated λ-ssDNA precursor^39^ was captured between SPHERO streptavidin coated polystyrene beads (4.82 μm) at 0.31 pN/nm trap stiffness, at 0.005% (w/v) bead concentration using the laminar flow cell, stretched and held at forces of 100 pN or higher in 25 mM Tris-HCl pH 7.5, 50 mM NaCl, 1 mM MgCl_2_ supplemented with 0.2 mg/ml BSA until the DNA strands were fully melted. The presence of ssDNA was verified by comparison with a built-in freely-jointed chain model. For most imaging conditions, ssDNA was held at forces between 10 and 20 pN, which corresponds to a 1.5-fold extension of B-form λ dsDNA. Beads and DNA were kept in PBS during the experiment.

For confocal imaging, three excitation wavelengths were used, 488 nm for AF488, 532 nm for mStrawberry and AF555, and 638 nm for AF647, with emission detected in three channels with a blue filter 512/25 nm, green filter 585/75 nm and red filter 640 LP, respectively. The imaging conditions were as follows. For the RAD51^AF488^/RPA^mStrawberry^ exchange assay: 5% blue laser power (3.01 μW), 7% green laser power (2.95 μW), 0.1 ms/pixel dwell-time, 100 nm pixel size, 5 s inter-frame wait time. For direct imaging of B^AF647^CDX2^AF555^, B^E144A/AF647^CDX2^AF555^ or B^AF647^C^E161A^DX2^AF555^ in the absence or presence of RAD51: 5% blue laser power (3.01 μW), 10% red laser power (3.23 μW), 0.1 ms/pixel dwell-time, 100 nm pixel size, 200 ms inter-frame wait time. For C-trap smFRET imaging of RAD51^AF555^ in the presence or absence of B^AF647^CDX2 or BCDX2^AF647^: 15% green laser power (6.30 μW), 0.1 ms/pixel dwell-time, 100 nm pixel size, 200 ms inter-frame wait time.

For RAD51^AF488^/RPA^mStrawberry^ exchange assays, λ ssDNA was pre-coated with 5 nM RPA^mStrawberry^ and transferred to a channel containing 50 nM RAD51^AF488^, 50 nM RAD51^C319S^ in the absence or presence of 10 nM wild-type BCDX2 in SM reaction buffer (50 mM Tris-HCl pH 7.5, 100 mM NaCl, 2 mM MgCl2 and 1 mM CaCl2 supplemented with 2 mM ATP and 0.2 mg/ml BSA) to monitor RAD51 filament assembly. To monitor RAD51 filament disassembly, after at least 15 min of growth, assembled RAD51^AF488^ filaments were transferred into a channel of SM reaction buffer lacking CaCl_2_ and ATP, and containing 5 nM RPA-mStrawberry.

For RAD51^AF488^/RPA^mStrawberry^ exchange assays with catalytic glutamate mutants, λ ssDNA was pre-coated with 5 nM RPA^mStrawberry^, and transferred to a channel containing 50 nM RAD51^AF488^ and 150 nM RAD51^C319S^ in the absence or presence of 40 nM BCDX2, B^E144A^CDX2, BC^E161A^DX2 or B^E144A^C^E161A^DX2 in SM reaction buffer. Higher total RAD51 and BCDX2 concentrations were used to increase the dynamic range of RAD51 filament assembly readout. For fluorescent BCDX2 binding ssDNA, 1 nM B^AF555^CDX2^AF647^, B^E144A/AF555^CDX2^AF647^ or B^AF555^C^E161A^DX2^AF647^ was monitored in the absence and presence of 25 nM RAD51^C319S^ in SM reaction buffer. For C-trap smFRET experiments, 5 nM RAD51^AF555^ alone or with 1 nM B^AF647^CDX2 or BCDX2^AF647^ was mixed in SM reaction buffer.

### Single-molecule analysis

For all experiments, real-time force and fluorescence data were exported from Bluelake HDF5 files and analysed using custom-written scripts in Pylake Python package. Statistical analyses and figure plotting were performed using GraphPad Prism 9.

For RAD51^AF488^/RPA^mStrawberry^ exchange assays, AF488 intensity was imported in Fiji^57^ and normalized to the background AF488 signal. Normalized AF488 signal changes over time were fitted with a single exponential function, y = A_max_(1-exp(-k*t)), to obtain assembly half-lives and global RAD51^AF488^ assembly rates. For analysis of B^AF647^CDX2^AF555^, B^E144A/AF647^CDX2^AF555^ or B^AF647^C^E161A^DX2^AF555^ binding kinetics, averaged AF555 fluorescence signals were plotted against time.

Forces were downsampled to 3 Hz for plotting. A worm-like chain (WLC) model for λ dsDNA was used as a reference for force-extension curve comparison. For reactions containing unlabelled RAD51 and b^AF647^CDX2^AF555^, b^E144A/AF647^CDX2^AF555^ or B^AF647^C^E161A^DX2^AF555^, force-time curves were used as a proxy for RAD51 filament assembly.

For apparent nucleation rate analysis, a custom script was used to quantify the AF488 or AF555 nucleation frequency from the kymographs showing fluorescence intensity increases with time. To quantify apparent nucleation rates, images were smoothed along the y-axis using a Savitzky-Golay filter (5 pixel window). A peak detection function was employed on the processed image along the same axis. The number of detected intensity peaks in each timeframe were fitted with a single exponential function y = A_ma_χ(1-exp(-k*t)), whereby k is the nucleation rate. Growth and disassembly rates of individual RAD51^AF488^ filaments were manually measured, and rates were calculated in Fiji.

For analysis of C-trap smFRET assays, AF555/AF647 intensities were averaged within a 3 px window along BCDX2/RAD51 binding events within the kymograph. FRET efficiencies were not calculated due to the presence of multiple fluorophores in RAD51 nuclei and overall high background for RAD51^AF555^. Instead, AF647 intensity was used as a proxy for FRET efficiency. To calculate background AF647 fluorescence (AF555 blead-through), we used AF647 intensity measured for RAD51^AF555^ clusters in the absence of labelled BCDX2. In the presence of B^AF647^CDX2 or BCDX2^AF647^, AF647-intensities were clearly identified above background and anti-correlated with AF555 intensity due to FRET.

### Hydrogen-deuterium exchange

Resource Q purified BCDX2 was gel filtrated on a Superdex 200 Increase 3.2/300 GL column (Cytiva) equilibrated in HMT buffer containing 100 mM NaCl on a Micro-kit equipped ÄKTA pure. In the BCDX2-ADP vs (BCDX2-ADP.AlFx or BCDX2- ADP.BeF_x_) comparative HDX-MS experiment, fractions containing BCDX2 were pooled and mixed with either ADP (0.5 mM ADP, 10 mM NaF), ADP.AlFx (0.5 mM ADP, 0.5 mM AlCl_3_, 10 mM NaF) or ADP.BeFx (0.5 mM ADP, 0.5 mM BeSO_4_, 10 mM NaF) to achieve a final concentration of 5 μM BCDX2. In the BCDX2-ADP.AlFx vs BCDX2-ADP.AlFx-ssDNA comparative HDX-MS experiment, ADP.AlFx (0.5 mM ADP, 0.5 mM AlCl_3_, 10 mM NaF) ± dN^12nt^ (15 μM) ssDNA was added to 5 μM BCDX2. Complexes (5 μL) were incubated with 40 μL of equivalent (ADP/ADP.AlFx/ADP.BeFx) deuterated (D_2_O) buffer and incubated for 3, 30, 300 and 3000 seconds in triplicate. The labelling reaction was quenched by adding chilled 2.4% (v/v) formic acid in 2 M guanidinium hydrochloride and immediately frozen in liquid nitrogen. Samples were stored at -80°C prior to analysis.

The quenched protein samples were rapidly thawed and subjected to proteolytic cleavage by pepsin followed by reversed phase HPLC separation. Briefly, the proteins were passed through an Enzymate BEH immobilized pepsin column (2.1 x 30 mm, 5 μm, Waters) at 200 μL/min for 2 min and the peptides trapped and desalted on a C18 trap column (Acquity BEH C18 Van-guard pre-column, 1.7 μm, 2.1 x 5 mm, Waters). Trapped peptides were subsequently eluted over 12 min using a 5-36% gradient of acetonitrile in 0.1% (v/v) formic acid at 40 μL/min. Peptides were separated on a reverse phase column (Acquity UPLC BEH C18 column 1.7 μm, 100 mm x 1 mm (Waters), and detected on a SYNAPT G2-Si HDMS mass spectrometer (Waters) acquiring over a m/z of 300 to 2000, with the standard electrospray ionization (ESI) source and lock mass calibration using [Glu1]-fibrino peptide B (50 fmol/μL). The mass spectrometer was operated at a source temperature of 80°C and a spray voltage of 3.0 kV. Spectra were collected in positive ion mode using MassLynx software.

Peptide identification was performed by MS^e^ using an identical gradient of increasing acetonitrile in 0.1% v/v formic acid over 12 min^58^. The resulting MS^e^ data were analysed using Protein Lynx Global Server software (Waters, UK) with an MS tolerance of 5 ppm.

Mass analysis of the peptide centroids was performed using DynamX software (Waters). Only peptides with a score >6.4 were considered. The first round of analysis and identification was performed automatically by the DynamX software; however, all peptides (deuterated and non-deuterated) were manually verified at every time point for the correct charge state, presence of overlapping peptides, and correct retention time. Deuterium incorporation was not corrected for back-exchange and represents relative, rather than absolute changes in deuterium levels. Changes in H/D amide exchange in any peptide may be due to a single amide or several amides within that peptide. All time points in this study were prepared at the same time and individual time points were acquired on the mass spectrometer on the same day.

### Bioinformatic analysis and molecular modelling

To highlight the conservation of catalytic glutamates and lysine fingers within the RAD51 paralog family, the amino acid sequences of human RAD51, RAD51B, RAD51C, RAD51D and XRCC2 were aligned using ClustalOmega^59^ and exported using ESPript3^60^. To highlight the conversation of putative arginine ssDNA binding residues, the human, chimp, mouse, rat, dog, zebrafish and chicken sequences for RAD51B, RAD51C, RAD51D and XRCC2 were aligned using ClustalOmega and exported using ESPript3.

All clinical VUS were extracted from Clinvar on 18/1/2023. To assess clinical VUS for effect on protein stability and folding, we extracted each subunit from the BCDX2-ADP.AlFx structure and *in silico* screened all missense mutations using PremPS^61^. We conservatively took only the highest-scoring hits (ΔΔG ≥ 2 kcal mol^-1^) and plotted them on structures of RAD51B, RAD51C, RAD51D and XRCC2. To assess protein-protein and protein-nucleotide interactions, we extracted hydrogen and ionic bonds within BCDX2-AlFx using the PDBSum server^62^ and plot them as a 2D interaction diagram. Non-bonded contacts for protein-protein interactions were not investigated. Supplementary Table 1 shows stratification of ClinVar VUS.

The atomic model of BCDX2 in the presence of ADP.AlFx was modified with MODELLER^63^ to add missing residues and loops, which were used for depicting exposed/protected regions in HDX-MS experiments. BCDX2 structure was modelled using a locally installed version of AlphaFold2^64^. Matchmaker in ChimeraX^65^ was used to align RAD51B^CTD^ of the AlphaFold model to RAD51-1 (PDB = 5H1B). A nucleotide triplet was manually appended to the 5’ end of ssDNA in the RAD51 pdb file to model RAD51C-ssDNA interactions.

### NS-EM sample preparation and data acquisition

The effect of BCDX2 on RAD51 filament formation was analysed as follows: Linear ssDNAs^66^ (4.5 μM, nucleotide concentration, 831 nt in length) were incubated with RAD51 (500 nM), ± BCDX2 (20 nM) in HMT buffer containing 100 mM NaCl and 1 mM ATP at 37°C for 30 minutes.

Samples containing BCDX2 in the presence of different nucleotides were prepared as follows. Resource Q purified BCDX2 was gel filtered on a Superdex 200 Increase 10/300 GL column (Cytiva) equilibrated in HMT buffer containing 100 mM NaCl on an ÄKTA pure. BCDX2 was diluted to 20 ng/μL in the presence of 1 mM ATP, 1 mM ADP or 1 mM ADP.BeFx (1 mM ADP, 1 mM BeSO4, 10 mM NaF).

Samples containing B^NTD^CDX2 were generated by diluting purified protein directly to 15 ng/μL into HMT buffer containing 100 mM NaCl and 1 mM ADP.BeFx.

Samples for NS-EM single particle analysis (SPA) of chymotrypsin-treated BCDX2 were diluted to 15 ng/μL in HMT buffer containing 0.5 mM ADP.BeFx.

Samples (4 μL) were then applied for 1 min to glow discharged (25 mA, 30 seconds) 400-mesh carbon-coated copper grids (EM Resolutions). The grids were sequentially stained in four separate 35 μL droplets of 2% (v/v) uranyl acetate for 5, 10, 15 and 20 seconds. Excess uranyl acetate was blotted away from the grid using Whatmann paper, allowed to air dry and stored before imaging.

Grids containing RAD51 filaments were imaged on a JEOL tungsten 1400FLASH TEM operating at 120 kV. A 2K Matataki Flash sCMOS camera was used to collect micrographs at a nominal magnification of 30,000X (5.85 Å pixel size). 631 micrographs were automatically acquired for each condition using SerialEM.

All other samples were imaged on a Tecnai LaB_6_ G^2^ Spirit TEM operating at 120 kV. A 2K Gatan Ultrascan 100 camera operated with Digital Micrograph was used to collect micrographs of BCDX2, B^NTD^CDX2 and chymotrypsin treated BCDX2 at a nominal magnification of 42,000X (2.4 Å pixel size). Micrographs were manually acquired with defocus values ranging from -0.8 to -1.5 μm.

### NS-EM data analysis

Fully automated scripts were written and performed in ImageJ to allow high throughput analyses of RAD51 filaments and lengths. A difference of Gaussians filter was applied to micrographs to enhance the contrast of RAD51 filaments, and curvilinear line analysis performed to automatically detect filaments and measure their lengths. Filaments were written out in a binary jpeg file and used to confirm suitable detection of filaments. In parallel, a trained crYOLO model was used to detect segments of RAD51 filaments, to provide another unbiased method to quantify RAD51 filament stimulation^67^. Statistical analyses and figure plotting were performed using GraphPad Prism 9.

For NS-EM SPA, micrographs were imported into Relion 3.1 or 4.0^70^, CTF parameters were calculated using CTFFIND4 and particles picked using a trained crYOLO^67^ or Topaz^69^ model. Particles were extracted and iteratively 2D classified (ignore CTF to first peak = yes, limit resolution E-step = 20 Å, additional arguments = --only-flip-phases, mask = 180 Å). The mobility of the RAD51B^CTD^ relative to B^NTD^CDX2 was monitored by manually aligning side orientations of 2D classes in Adobe Photoshop.

### Cryo-EM sample preparation and data acquisition

For the BCDX2-ADP.BeF_x_ sample, Resource Q purified BCDX2 was gel filtered using a Superdex 200 Increase 3.2/300 column (Cytiva) equilibrated in HMT buffer containing 100 mM NaCl on a Micro-kit equipped ÄKTA pure. Fractions containing BCDX2 were incubated for 5 min at RT with 0.5 mM ADP.BeF_x_ (0.5 mM ADP, 0.5 mM BeSO_4_ and 10 mM NaF) and crosslinked with 0.005% glutaraldehyde for 30 min at RT in the presence of 0.0005% Tween20. The sample was quenched with 50 mM Tris-HCl pH 7.5 for 15 min at room temperature. The final sample was 0.25 mg/mL (1.7 μM). An aliquot (4 μL) was applied to glow discharged (Quorum Emitech K100X, 25 mA, 60 seconds) UltrAuFoil R2/2 Au200 grids, incubated for 5 s, double sided blotted for 2 s and plunge frozen in liquid ethane using a Vitrobot (4°C, 95% humidity). The cryo-EM dataset was collected on a FEI Titan Krios microscope operating at 300 kV, using a K2 summit direct electron detector camera (Gatan) operating with 1.08 Å per pixel and 6 electrons per pixel per second. Movies were collected using EPU (ThermoFisher), each approximately 9 seconds, dose-fractioned into 30 frames and containing a total dose of 47 electrons per Å^2^. 12,555 movies were collected with zero tilt, and a further 11,894 movies with a 20° tilt.

BCDX2-ADP.AlFx-ssDNA complexes were prepared as follows. An equal volume of Resource Q purified BCDX2, stored in HGMT buffer with 300 mM NaCl, was diluted with HGMT buffer such that the final concentration of NaCl reached 150 mM. 0.5 mM ADP.AlFx (0.5 mM ADP, 0.5 mM AlCl_3_ and 10 mM NaF) was added and incubated for 5 min. Subsequently, a 4.5-fold molar excess of FAM-dN^12nt^ ssDNA was mixed with the sample for 10 min, followed by 10 min crosslinking with 0.005% glutaraldehyde at RT. The sample was quenched by the addition of 50 mM Tris-HCl pH 7.5. The sample was gel filtered using a Superdex 200 Increase 3.2/300 column (Cytiva) equilibrated in HMT buffer containing 100 mM NaCl and 0.5 mM ADP.AlFx on a Micro-kit equipped ÄKTA pure. UV monitoring at 280 nm and 495 nm allowed simultaneous detection of protein and FAM-dN^12nt^. Concentrations of protein-DNA complexes were measured using Bradford reagent and diluted to 0.25 mg/mL (1.7 μM) with final detergent concentrations of 0.00075% Tween-20 and 0.075 mM CHAPSO. A sample (4 μL) was applied to glow discharged (Quorum Emitech K100X, 45 mA, 60 seconds) UltrAuFoil R2/2 Au200 grids, incubated for 5 s, double side blotted for 2 s and plunge frozen in liquid ethane using a Vitrobot (4°C, 95% humidity). The cryo-EM dataset was collected on a FEI Titan Krios microscope operating at 300 kV, using a K3 summit direct electron detector camera (Gatan) operating in correlated-double sampling mode with 0.85 Å per pixel and 14 electrons per pixel per second. Movies were collected using EPU (ThermoFisher), each approximately 2.78 seconds, dose-fractioned into 50 frames and containing a total dose of 53.2 electrons per Å^2^. A total of 35,305 movies were collected with no tilt.

### Cryo-EM data analysis

All single particle analyses were performed within Relion 4.0^70^. The movies were corrected for drift and dose-weighted using MOTIONCOR2^71^, and subsequent contrast transfer (CTF) parameters were measured using CTFFIND4^68^. For the BCDX2-ADP.BeFx dataset, the dAst (amount of astigmatism) value for CTFFIND4 was set to 100 and 1000 Å for the non-tilted and tilted data collection, respectively. Particles were picked automatically using Topaz^69^ from the non-tilt dataset, extracted (FOM = -1), yielding 4,603,811 particles that were iteratively 2D and 3D classified, leading to an initial 3D model. Particles across both the tilted and non-tilted datasets were picked with Topaz and extracted (FOM = 1, 2.16 Å/px, binning = 2, box size = 128 Å^2^), resulting in a total of 4,528,940 particles. The initial 3D model was low pass filtered to 50 Å and used as an initial model for direct 3D classification (10 classes, 150 Å mask diameter, T = 6). Promising 3D classes were selected, and further 3D classified (both unmasked with alignment followed by masked without alignment). The resulting 375,855 particles were refined using a reference mask, leading to a resolution of 4.4 Å. These particles were unbinned (box size = 256 Å), further 3D classified (without alignment) and refined, leading to a resolution of 4.1 Å. Bayesian polishing was performed with optimised parameters for the non-tilted and titled datasets, followed by CTF refinement (with corrective fitting for defocus and astigmatism). The particles were refined, again with a reference mask, leading to a resolution of 3.7 Å. A final 3D classification without alignment and with a reference mask (2 classes, mask diameter = 220 Å, T = 10) and subsequent refinement resolved a 3.6 Å map. Particles were further polished and CTF parameters refined (CTF parameter fitting, anisotropic magnification, beam tilt and trefoil), yielding a final resolution (measured at FSC = 0.143)^72^ equal to 3.4 Å.

For the BCDX2-ADP.AlFx-ssDNA dataset, 25,886,382 particles were picked automatically using a trained model with Topaz, extracted using a figure of merit (FOM) threshold equal to 1 and a box size of 100 Å^2^ (3.06 Å/px, 3.6-fold down-sampled). Two rounds of 2D classification were performed, yielding 7,376,996 particles. The map of BCDX2-ADP.BeFx generated from the previous data set, low pass filtered to 40 Å, was used as an initial model for 3D classification (3 classes, 180 Å mask, T=4). 3,934,738 particles were re-extracted (1.275 Å/px, 1.5-fold) and refined, leading to resolutions between 2.8 - 2.9 Å across three subsets. To achieve the consensus refinement (BCDX2-ADP.AlFx), particles were polished (and re-extracted, 1.02 Å/px, 1.2-fold), refined, masked and 3D classified without particle alignment (3 classes, 240 Å mask, T=20). The three classes were refined, yielding resolutions equal to 2.4 Å (1,371,033 particles), 3.2 Å (1,465,570 particles) and 3.0 Å (1,098,043 particles). CTF values for the particles yielding the 2.4 Å density were refined (per particle refinement, beam tilt, trefoil, 4^th^ order aberrations), and particles further polished (re-extract, 0.95625 Å/px) and refined leading to a masked resolution equal to 2.2 Å. Further classification yielded no improvements in resolution or separation of heterogeneity. Attempts to resolve the RAD51B^CTD^ by using a *de novo* initial model early in classification (i.e., not the BCDX2-ADP.BeFx structure), or by focussed classification of empty space where RAD51B^CTD^ is expected, yielded no extra density. RAD51B^CTD^ is likely not observed due to high flexibility and a relatively low affinity to RAD51C^CTD^. To achieve the BCDX2-ADP.AlFx-ssDNA refinement, a mask was generated around the L1 and L2 DNA binding loops of RAD51C, RAD51D and XRCC2 using UCSF ChimeraX^65^, and used to subtract density from 3,934,738 particles. Particles were 3D classified (30 classes, 80 Å mask, T=10), and each 3D class reverted to the original particles for refinement. Refinements were manually inspected, and maps of interest further CTF refined and polished. Only one class contained density for ssDNA, which resolved to a final resolution equal to 2.9 Å. Due to high variability in local resolution, the refinement was post-processed with DeepEMhancer^73^.

### Atomic model building

All model building was achieved using Phenix^74^, ISOLDE and COOT^75^. The sharpened BCDX2-ADP.BeFx 3.4 Å map was imported into Phenix, alongside the AlphaFold^64^ predictions of RAD51B, RAD51C, RAD51D and XRCC2. Dock and rebuild was used within Phenix to place the four proteins into the cryo-EM density. Once in the correct position, ATP and ADP with their coordinating magnesium ions were manually fit into density within the active site of each subunit in COOT. The atomic model was iteratively real space refined in Phenix and manually modified in COOT. The resulting BCDX2-ADP.BeFx model was used as an initial model for building into the 2.2 Å BCDX2-ADP.AlFx map, which was iteratively real space refined in Phenix and manually modified in COOT. The 2.2 Å BCDX2-ADP.AlFx density was doused with water using Phenix, which were manually inspected and verified in COOT. All figures containing atomic structures were generated using ChimeraX^65^.

### Statistics and reproducibility

Statistical analyses were performed using GraphPad Prism 9. Normally distributed data were compared using two-tailed unpaired t-tests whereas non-normally distributed data were compared using two-tailed Mann-Whitney u-tests. Differences were considered statistically significant when p<0.05. Reported n values refer to independent experiments for fluorescence anisotropy, ATPase, HPLC and bulk FRET ssDNA binding assays. For NS-EM analyses, n values refer to number of micrographs. For single molecule analyses, n values refer to either the number of ssDNA molecules or fluorescent proteins/clusters.

## Extended Data

**Extended Data Table 1:** Cryo-EM data collection, refinement and validation statistics

	BCDX2-ADP.AlFx (EMDB-17206) (PDB 8OUZ)	BCDX2- ADP.AlFx-ssDNA (EMDB-17207)	BCDX2-ADP.BeFx (EMDB-17205) (PDB 8OUY)
**Data collection and processing**			
Magnification	105,000	105,000	130,000
Voltage (kV)	300	300	300
Electron exposure (e–/Å^2^)	53.2	53.2	47
Defocus range (μm)	-0.75 - -2.25	-0.75 - -2.25	-1.5 - -3.5
Pixel size (Å)	0.85	0.85	1.08
Symmetry imposed	C1	C1	C1
Initial particle images (no.)	25,886,382	25,886,382	4,528,940
Final particle images (no.)	1,371,033	241,626	87,572
Map resolution (Å)	2.2	2.9	3.4
FSC threshold	0.143	0.143	0.143
Map resolution range (Å)	2.2-3.3	2.8-6.3	3.4-5.2
**Refinement**			
Initial model (PDB code)	8OUY		AlphaFold
Model resolution (Å)	2.3		3.5
FSC threshold	0.5		0.5
Map sharpening *B* factor (Å^2^)	-30		-104.921
Model composition			
Non-hydrogen atoms	7449		7312
Protein residues	924		925
Ligands	MG: 3		MG : 3
	ADP: 1		A DP: 1
	ATP: 2		ATP: 2
*B* factors (Å^2^)			
Protein	48.93		54.18
Ligand	37.33 (water = 42.58)		49.77
R.m.s. deviations			
Bond lengths (Å)	0.003		0.02
Bond angles (°)	0.561 (0)		0.522 (1)
Validation			
MolProbity score	1.44		1.55
Clashscore	4.35		4.62
Poor rotamers (%)	0.00		0.00
Ramachandran plot			
Favored (%)	96.58		95.48
Allowed (%)	3.42		4.52
Disallowed (%)	0.00		0.00

## Supplementary Material

Supplementary Data Table 1

## Data Availability

Cryo-EM density maps and atomic models of BCDX2 have been deposited in the Electron Microscopy Data Bank (EMDB) and Protein Database (PDB). Accession codes are as follows: BCDX2-ADP.AlFx (EMDB = 17206, PDB = 8OUZ), BCDX2-ADP.AlFx-ssDNA (EMDB = 17207) and BCDX2-ADP.BeFx (EMDB = 17205, PDB = 8OUY). The atomic model of RAD51 was obtained from the PDB (accession = 5H1B). All other data and materials reported here are available on request.

## References

[1] Venkitaraman AR (2014). Cancer suppression by the chromosome custodians, BRCA1 and BRCA2. Science.

[2] Golmard L (2013). Germline mutation in the *RAD51B* gene confers predisposition to breast cancer. BMC Cancer.

[3] Song H (2015). Contribution of germline mutations in the *RAD51B, RAD51C* and *RAD51D* genes to ovarian cancer in the population. J Clin Oncol.

[4] Meindl A (2010). Germline mutations in breast and ovarian cancer pedigrees establish *RAD51C* as a human cancer susceptibility gene. Nat Genet.

[5] Pelttari LM (2011). *RAD51C* is a susceptibility gene for ovarian cancer. Hum Mol Genet.

[6] Loveday C (2011). Germline mutations in *RAD51D* confer susceptibility to ovarian cancer. Nat Genet.

[7] Prakash R (2022). Homologous recombination-deficient mutation cluster in tumor suppressor *RAD51C* identified by comprehensive analysis of cancer variants. Proc Natl Acad Sci USA.

[8] Park DJ (2012). Rare mutations in *XRCC2* increase the risk of breast cancer. Am J Hum Genet.

[9] Bhattacharya D (2022). RAD51 paralogs: Expanding roles in replication stress responses and repair. Curr Op Pharmacol.

[10] Bonilla B, Hengel SR, Grundy MK, Bernstein KA (2020). *RAD51* gene family structure and function. Annu Rev Genet.

[11] Park JY (2016). Complementation of hypersensitivity to DNA interstrand crosslinking agents demonstrates that *XRCC2* is a Fanconi anaemia gene. J Med Genet.

[12] Vaz F (2010). Mutation of the *RAD51C* gene in a Fanconi anemia-like disorder. Nat Genet.

[13] Shamseldin HE, Elfaki M, Alkuraya FS (2012). Exome sequencing reveals a novel Fanconi group defined by *XRCC2* mutation. J Med Genet.

[14] Berti M (2020). Sequential role of RAD51 paralog complexes in replication fork remodeling and restart. Nat Commun.

[15] Garcin EB (2019). Differential requirements for the RAD51 paralogs in genome repair and maintenance in human cells. PLoS Genet.

[16] Lin ZG, Kong HZ, Nei M, Ma H (2006). Origins and evolution of the *recA/RAD51* gene family: Evidence for ancient gene duplication and endosymbiotic gene transfer. Proc Natl Acad Sci U S A.

[17] Baumann P, Benson FE, West SC (1996). Human RAD51 protein promotes ATP-dependent homologous pairing and strand transfer reactions *in vitro*. Cell.

[18] Benson FE, Stasiak A, West SC (1994). Purification and characterisation of the human RAD51 protein, an analogue of *E. coli* RecA. EMBO J.

[19] Sung P, Robberson DL (1995). DNA strand exchange mediated by a RAD51-ssDNA nucleoprotein filament with polarity opposite to that of RecA. Cell.

[20] Xu J (2017). Cryo-EM structures of human RAD51 recombinase filaments during catalysis of DNA-strand exchange. Nat Struct Mol Biol.

[21] Lee JY (2015). Base triplet stepping by the Rad51/RecA family of recombinases. Science.

[22] Chen ZC, Yang HJ, Pavletich NP (2008). Mechanism of homologous recombination from the RecA-ssDNA/dsDNA structures. Nature.

[23] Short JM (2016). High-resolution structure of the presynaptic RAD51 filament on single-stranded DNA by electron cryo-microscopy. Nucl Acids Res.

[24] Sung P, Klein H (2006). Mechanism of homologous recombination: mediators and helicases take on regulatory functions. Nat Rev Mol Cell Biol.

[25] Chen PL (1998). The BRC repeats in BRCA2 are critical for RAD51 binding and resistance to methyl methanesulfonate treatment. Proc Natl Acad Sci USA.

[26] Davies AA (2001). Role of BRCA2 in control of the RAD51 recombination and DNA repair protein. Mol Cell.

[27] Yang HJ (2002). BRCA2 function in DNA binding and recombination from a BRCA2-DSS1-ssDNA structure. Science.

[28] Pellegrini L (2002). Insights into DNA recombination from the structure of a RAD51-BRCA2 complex. Nature.

[29] Jensen RB, Carreira A, Kowalczykowski SC (2010). Purified human BRCA2 stimulates RAD51-mediated recombination. Nature.

[30] Thorslund T (2010). The breast cancer tumour suppressor BRCA2 promotes the specific targeting of RAD51 to single-stranded DNA. Nat Struct Mol Biol.

[31] Esashi F (2005). CDK-dependent phosphorylation of BRCA2 as a regulatory mechanism for recombinational repair. Nature.

[32] Esashi F, Galkin VE, Yu X, Egelman EH, West SC (2007). Stabilisation of RAD51 nucleoprotein filaments by the C-terminal region of BRCA2. Nat Struct Mol Biol.

[33] Shahid T (2014). Structure and mechanism of action of the BRCA2 breast cancer tumour suppressor. Nature Struct Mol Biol.

[34] Xia B (2006). Control of BRCA2 cellular and clinical functions by a nuclear partner, PALB2. Mol Cell.

[35] Zhang F (2009). PALB2 links BRCA1 and BRCA2 in the DNA-damage response. Curr Biol.

[36] Buisson R (2010). Cooperation of breast cancer proteins PALB2 and piccolo BRCA2 in stimulating homologous recombination. Nat Struct Mol Biol.

[37] Masson J-Y (2001). Identification and purification of two distinct complexes containing the five RAD51 paralogs. Genes Dev.

[38] Liu J (2011). Rad51 paralogues Rad55-Rad57 balance the antirecombinase Srs2 in Rad51 filament formation. Nature.

[39] Belan O (2021). Single-molecule analysis reveals cooperative stimulation of Rad51 filament nucleation and growth by mediator proteins. Mol Cell.

[40] Roy U (2021). The Rad51 paralog complex Rad55-Rad57 acts as a molecular chaperone during homologous recombination. Mol Cell.

[41] Amunugama R (2012). RAD51 protein ATP cap regulates nucleoprotein filament stability. J Biol Chem.

[42] Miller KA, Sawicka D, Barsky D, Albala JS (2004). Domain mapping of the RAD51 paralog protein complexes. Nucl Acids Res.

[43] Yang H, Zhou C, Dhar A, Pavletich NP (2020). Mechanism of strand exchange from RecA-DNA synaptic and D-loop structures. Nature.

[44] Landrum MJ (2018). ClinVar: improving access to variant interpretations and supporting evidence. Nucl Acids Res.

[45] Lacabanne D (2020). ATP analogues for structural investigations: Case studies of a DnaB helicase and an ABC transporter. Molecules.

[46] Evans R (2023). Protein complex prediction with AlphaFold-Multimer. BioxRiv.

[47] Anand R (2022). HELQ is a dual-function DSB repair enzyme modulated by RPA and RAD51. Nature.

[48] Hegner M, Smith SB, Bustamante C (1999). Polymerization and mechanical properties of single RecA-DNA filaments. Proc Natl Acad Sci USA.

[49] Baumann P, West SC (1997). The human RAD51 protein: polarity of strand transfer and stimulation by hRP-A. EMBO J.

[50] Subramanyam S, Ismail M, Bhattacharya I, Spies M (2016). Tyrosine phosphorylation stimulates activity of human RAD51 recombinase through altered nucleoprotein filament dynamics. Proc Natl Acad Sci U-S-A.

[51] Sano K, Maeda K, Oki M, Maeda Y (2002). Enhancement of protein expression in insect cells by a lobster tropomyosin cDNA leader sequence. FEBS Lett.

[52] Weissmann F (2016). biGBac enables rapid gene assembly for the expression of large multisubunit protein complexes. Proc Natl Acad Sci U-S-A.

[53] Yin J (2005). Genetically encoded short peptide tag for versatile protein labeling by Sfp phosphopantetheinyl transferase. Proc Natl Acad Sci U-S-A.

[54] Hitchman RB, Siaterli EA, Nixon CP, King LA (2007). Quantitative realtime PCR for rapid and accurate titration of recombinant baculovirus particles. Biotechnol Bioeng.

[55] Theile CS (2013). Site-specific N-terminal labeling of proteins using sortase-mediated reactions. Nat Protoc.

[56] Liu Y, Tarsounas M, O’Regan P, West SC (2007). Role of RAD51C and XRCC3 in genetic recombination and DNA repair. J Biol Chem.

[57] Schindelin J (2012). Fiji: an open-source platform for biological-image analysis. Nat Methods.

[58] Silva JC (2005). Quantitative proteomic analysis by accurate mass retention time pairs. Anal Chem.

[59] Sievers F (2011). Fast, scalable generation of high-quality protein multiple sequence alignments using Clustal Omega. Mol Syst Biol.

[60] Robert X, Gouet P (2014). Deciphering key features in protein structures with the new ENDscript server. Nucl Acids Res.

[61] Chen Y (2020). PremPS: Predicting the impact of missense mutations on protein stability. PLoS Comput Biol.

[62] Laskowski RA, Jablonska J, Pravda L, Varekova RS, Thornton JM (2018). PDBsum: Structural summaries of PDB entries. Protein Sci.

[63] Webb B, Sali A (2016). Comparative Protein Structure Modeling Using MODELLER. Curr Protoc Bioinformatics.

[64] Jumper J (2021). Highly accurate protein structure prediction with AlphaFold. Nature.

[65] Pettersen EF (2021). UCSF ChimeraX: Structure visualization for researchers, educators, and developers. Protein Sci.

[66] Ho HN, West SC (2022). Generation of double Holliday junctiom DNAs and their dissolution/resolution within a chromatin context. Proc Natl Acad Sci-U-S-A.

[67] Wagner T (2019). SPHIRE-crYOLO is a fast and accurate fully automated particle picker for cryo-EM. Commun Biol.

[68] Rohou A, Grigorieff N (2015). CTFFIND4: Fast and accurate defocus estimation from electron micrographs. J Struct Biol.

[69] Bepler T (2019). Positive-unlabeled convolutional neural networks for particle picking in cryo-electron micrographs. Nat Methods.

[70] Kimanius D, Dong L, Sharov G, Nakane T, Scheres SHW (2021). New tools for automated cryo-EM single-particle analysis in RELION-4.0. Biochem J.

[71] Zheng SQ (2017). MotionCor2: anisotropic correction of beam-induced motion for improved cryo-electron microscopy. Nat Methods.

[72] Rosenthal PB, Henderson R (2003). Optimal determination of particle orientation, absolute hand, and contrast loss in single-particle electron cryomicroscopy. J Mol Biol.

[73] Sanchez-Garcia R (2021). DeepEMhancer: a deep learning solution for cryo-EM volume post-processing. Commun Biol.

[74] Liebschner D (2019). Macromolecular structure determination using X-rays, neutrons and electrons: recent developments in Phenix. Acta Crystallogr D Struct Biol.

[75] Casanal A, Lohkamp B, Emsley P (2020). Current developments in Coot for macromolecular model building of Electron Cryo-microscopy and Crystallographic Data. Protein Sci.

