# Supplementary material for: Structure and function of the RAD51B-RAD51C-RAD51D-XRCC2 tumour suppressor
Source: Nature. Author manuscript; Available in PMC 2023 Jul 20. (PMC7614784; doi:10.1038/s41586-023-06179-1)
Supplement: Supplementary Data Table 1 [file EMS177404-supplement-Supplementary_Data_Table_1.docx]

**Supplementary Data Table 1: Pathogenic and missense VUS mutations in BCDX2**

| **Missense mutation** | **ClinVar accession code** | **Effect** |
| --- | --- | --- |
| **RAD51B** | | |
| Y68C | VCV000830255 | RAD51C (H-bond) |
| **RAD51C** | | |
| L14S | VCV001738651 | Predicted monomer instability |
| F17C | VCV000825447 | Predicted monomer instability |
| F17S | VCV000632964 | Predicted monomer instability |
| P18S | VCV000232581 | RAD51B (H-bond) |
| P18Q | VCV001517495 | RAD51B (H-bond) |
| P18R | VCV000945688 | RAD51B (H-bond) |
| P18L | VCV000480491 | RAD51B (H-bond) |
| L19P | VCV000655726 | Predicted monomer instability |
| V23G | VCV000830275 | Predicted monomer instability |
| R24G | VCV000924763 | RAD51C (H-bond), RAD51D (H-bond, ionic) |
| R24W | VCV000241776 | RAD51C (H-bond), RAD51D (H-bond, ionic) |
| R24L | VCV000421982 | RAD51C (H-bond), RAD51D (H-bond, ionic) |
| R24Q | VCV000229691 | RAD51C (H-bond), RAD51D (H-bond, ionic) |
| L27P | VCV000140837 | Predicted monomer instability |
| Q33H | VCV001425447 | XRCC2 (H-bond) |
| Q33H | VCV000823598 | XRCC2 (H-bond) |
| L38P | VCV000538768 | Predicted monomer instability |
| E40K | VCV000188123 | RAD51D (Ionic) |
| E40G | VCV000538767 | RAD51D (Ionic) |
| E40D | VCV001391616 | RAD51D (Ionic) |
| I52K | VCV001717849 | Predicted monomer instability |
| I52R | VCV001479139 | Predicted monomer instability |
| K84M | VCV001792504 | RAD51D (H-bond) |
| K84R | VCV001386523 | RAD51D (H-bond) |
| K84N | VCV000186362 | RAD51D (H-bond) |
| C85R | VCV000471440 | RAD51D (H-bond) |
| C85G | VCV000409853 | RAD51D (H-bond) |
| C85Y | VCV000484730 | RAD51D (H-bond) |
| A87S | VCV000844573 | RAD51D (H-bond) |
| A87T | VCV000821547 | RAD51D (H-bond) |
| A87E | VCV000631280 | RAD51D (H-bond) |
| L88V | VCV000538781 | RAD51D (H-bond) |
| I100K | VCV000630758 | Predicted monomer instability |
| T102N | VCV000960831 | Predicted monomer instability |
| C104R | VCV001098889 | Predicted monomer instability |
| I110S | VCV000490123 | Predicted monomer instability |
| L111P | VCV001730304 | Predicted monomer instability |
| V115A | VCV000654903 | Predicted monomer instability |
| C124G | VCV000928155 | Predicted monomer instability |
| C124S | VCV000496503 | Predicted monomer instability |
| C124S | VCV001359378 | Predicted monomer instability |
| P127S | VCV000538782 | ADP (RAD51C, Non-bonded) |
| P127T | VCV000142086 | ADP (RAD51C, Non-bonded) |
| P127R | VCV001719290 | ADP (RAD51C, Non-bonded) |
| G128R | VCV000246222 | ADP (RAD51C, H-bond) |
| G128D | VCV000490126 | ADP (RAD51C, H-bond) |
| G128A | VCV000482169 | ADP (RAD51C, H-bond) |
| V129I | VCV000478610 | ADP (RAD51C, Non-bonded) |
| V129A | VCV000233792 | ADP (RAD51C, Non-bonded) |
| G130R | VCV001098891 | ADP (RAD51C, H-bond) |
| G130A | VCV000928939 | ADP (RAD51C, H-bond) |
| K131Q | VCV001365945 | ADP (RAD51C, H-bond) |
| K131I | VCV000664529 | ADP (RAD51C, H-bond) |
| T132P | VCV000996829 | ADP (RAD51C, H-bond) |
| T132I | VCV000232015 | ADP (RAD51C, H-bond) |
| T132R | VCV000182834 | ADP (RAD51C, H-bond) |
| Q133K | VCV000233936 | ADP (RAD51C, H-bond) |
| Q133E | VCV000230813 | ADP (RAD51C, H-bond) |
| Q133R | VCV001098892 | ADP (RAD51C, H-bond) |
| L134S | VCV000538772 | Predicted monomer instability |
| C135R | VCV000241771 | Predicted monomer instability |
| M136R | VCV001438265 | Predicted monomer instability |
| V140E | VCV000920047 | Predicted monomer instability |
| V142G | VCV001739166 | Predicted monomer instability |
| I144T | VCV000142840 | Predicted monomer instability |
| F148S | VCV000220560 | Predicted monomer instability |
| V156G | VCV001098895 | Predicted monomer instability |
| V156D | VCV000825091 | Predicted monomer instability |
| F157C | VCV000478611 | Predicted monomer instability |
| I158T | VCV000478578 | Predicted monomer instability |
| T160K | VCV001743152 | Predicted monomer instability |
| E161Q | VCV000490128 | ATP (hydrolysis) |
| E161V | VCV000496504 | ATP (hydrolysis) |
| E161D | VCV000565947 | ATP (hydrolysis) |
| F164S | VCV000409845 | Predicted monomer instability |
| R168G | VCV000825386 | ADP (RAD51C, H-bond) |
| L172P | VCV001171792 | Predicted monomer instability |
| C176P | VCV001746438 | Predicted monomer instability |
| L180R | VCV000484760 | Predicted monomer instability |
| D202G | VCV000409847 | RAD51B (Ionic) |
| D202E | VCV000921191 | RAD51B (Ionic) |
| I204N | VCV001751727 | Predicted monomer instability |
| I208V | VCV000241773 | RAD51B (H-bond) |
| I208S | VCV001009535 | Predicted monomer instability, RAD51B (H-bond) |
| Y210C | VCV000826306 | RAD51B (H-bond) |
| C213S | VCV001522818 | Predicted monomer instability |
| L219S | VCV000232604 | Predicted monomer instability |
| L226P | VCV000486269 | Predicted monomer instability |
| V236D | VCV000538760 | Predicted monomer instability |
| L238P | VCV001376815 | Predicted monomer instability |
| L238R | VCV000409866 | Predicted monomer instability |
| V239A | VCV000826885 | Predicted monomer instability |
| V239E | VCV000421575 | Predicted monomer instability |
| I240T | VCV000241777 | Predicted monomer instability |
| V241A | VCV000484728 | Predicted monomer instability |
| D242Y | VCV001335285 | ADP (RAD51C, H-bond) |
| D242N | VCV000231486 | ADP (RAD51C, H-bond) |
| D242V | VCV001444549 | ADP (RAD51C, H-bond) |
| D242G | VCV000927198 | ADP (RAD51C, H-bond) |
| F246C | VCV001758612 | Predicted monomer instability |
| D253G | VCV000409850 | RAD51B (H-bond) |
| R258G | VCV000480518 | ssDNA binding |
| R258C | VCV000142453 | ssDNA binding |
| R258L | VCV000657073 | ssDNA binding |
| R258P | VCV000645806 | ssDNA binding |
| R258H | VCV000006822 | ssDNA binding |
| L265P | VCV000480945 | Predicted monomer instability |
| Q267H | VCV000959677 | RAD51D (H-bond) |
| M269T | VCV001761873 | Predicted monomer instability |
| L278S | VCV000482174 | Predicted monomer instability |
| H307Y | VCV000921160 | ATP (RAD51D, H-bond) |
| H307R | VCV000918247 | ATP (RAD51D, H-bond) |
| H307Q | VCV001491316 | ATP (RAD51D, H-bond) |
| I311K | VCV001443174 | Predicted monomer instability |
| I314T | VCV001472587 | Predicted monomer instability |
| F315C | VCV001312079 | Predicted monomer instability |
| L326W | VCV000965548 | Predicted monomer instability |
| L326S | VCV000823473 | Predicted monomer instability |
| K328E | VCV000492403 | ATP (RAD51D, H-bond) |
| K328T | VCV001768350 | ATP (RAD51D, H-bond) |
| K328N | VCV001718432 | ATP (RAD51D, H-bond) |
| S329L | VCV000566789 | ATP (RAD51D, Non-bonded) |
| P330S | VCV000471454 | RAD51D (H-bond), ATP (RAD51D, Non-bonded) |
| P330R | VCV000993148 | RAD51D (H-bond), ATP (RAD51D, Non-bonded) |
| P330L | VCV000577849 | RAD51D (H-bond), ATP (RAD51D, Non-bonded) |
| S331N | VCV001473818 | ATP (RAD51D, H-bond) |
| S331I | VCV000186085 | ATP (RAD51D, H-bond) |
| S331R | VCV000863234 | ATP (RAD51D, H-bond) |
| Q332K | VCV000823577 | ATP (RAD51D, Non-bonded) |
| K333E | VCV001768787 | ATP (RAD51D, Non-bonded) |
| K333R | VCV001768811 | ATP (RAD51D, Non-bonded) |
| K333N | VCV001039500 | ATP (RAD51D, Non-bonded) |
| E334K | VCV000633385 | ATP (RAD51D, H-bond) |
| F339S | VCV000409861 | Predicted monomer instability |
| I341V | VCV000851867 | ADP (RAD51C, Non-bonded) |
| F346S | VCV000490118 | Predicted monomer instability |
| **RAD51D** | | |
| R5S | VCV001511764 | RAD51C (Ionic bond) |
| R5K | VCV000917702 | RAD51C (Ionic bond) |
| R5W | VCV000233989 | RAD51C (Ionic bond) |
| L20R | VCV000472613 | Predicted monomer instability |
| I25N | VCV000926315 | Predicted monomer instability |
| L31P | VCV001766497 | Predicted monomer instability |
| V39G | VCV000565429 | Predicted monomer instability |
| L53P | VCV001508112 | Predicted monomer instability |
| S62W | VCV000187224 | XRCC2 (H-bond) |
| S62L | VCV000185036 | XRCC2 (H-bond) |
| S62P | VCV001781304 | XRCC2 (H-bond) |
| P64S | VCV000371900 | Predicted monomer instability |
| V66L | VCV000419663 | XRCC2 (H-bond) |
| V66M | VCV000141578 | XRCC2 (H-bond) |
| N67K | VCV001471055 | XRCC2 (H-bond) |
| N67S | VCV000630041 | XRCC2 (H-bond) |
| N67D | VCV001465033 | XRCC2 (H-bond) |
| G68S | VCV000410548 | XRCC2 (H-bond) |
| A78S | VCV000803385 | Predicted monomer instability |
| L84H | VCV000138878 | Predicted monomer instability |
| L89P | VCV000567175 | Predicted monomer instability |
| T89R | VCV000803384 | Predicted monomer instability |
| L93R | VCV000926961 | Predicted monomer instability |
| S93P | VCV000372057 | Predicted monomer instability |
| A96T | VCV000138880 | Predicted monomer instability |
| R100Q | VCV000492418 | Predicted monomer instability |
| G101A | VCV000492419 | Predicted monomer instability |
| V102G | VCV000539850 | Predicted monomer instability |
| A103V | VCV000548805 | Predicted monomer instability |
| R108L | VCV001210161 | Predicted monomer instability |
| R108C | VCV000425128 | Predicted monomer instability |
| L109F | VCV001051075 | Predicted monomer instability |
| G110V | VCV001729936 | ATP (RAD51D, Non-bonded) |
| G110A | VCV000229820 | ATP (RAD51D, Non-bonded) |
| G110D | VCV000127887 | ATP (RAD51D, Non-bonded) |
| S111R | VCV000583034 | ATP (RAD51D, H-bond) |
| S111N | VCV000187017 | ATP (RAD51D, H-bond) |
| S111G | VCV000836729 | ATP (RAD51D, H-bond) |
| G112A | VCV000234081 | ATP (RAD51D, H-bond) |
| G112D | VCV000142955 | ATP (RAD51D, H-bond) |
| G112S | VCV000627674 | ATP (RAD51D, H-bond) |
| K113N | VCV000185853 | ATP (RAD51D, H-bond) |
| K113E | VCV001171377 | ATP (RAD51D, H-bond) |
| T114I | VCV000945947 | ATP (RAD51D, H-bond) |
| T114P | VCV001496477 | ATP (RAD51D, H-bond) |
| Q115H | VCV000643598 | RAD51C (H-bond), ATP (RAD51D, H-bond) |
| Q115L | VCV001731537 | RAD51C (H-bond), ATP (RAD51D, H-bond) |
| Q115R | VCV000934207 | RAD51C (H-bond), ATP (RAD51D, H-bond) |
| V132D | VCV000539861 | Predicted monomer instability |
| V135E | VCV000657311 | Predicted monomer instability |
| S137F | VCV001493231 | RAD51C (H-bond) |
| S137A | VCV000472606 | RAD51C (H-bond) |
| R145H | VCV000187149 | ATP (RAD51D, Non-bonded) |
| R145L | VCV000924363 | ATP (RAD51D, Non-bonded) |
| R145C | VCV000231938 | ATP (RAD51D, Non-bonded) |
| L146H | VCV000648875 | Predicted monomer instability, RAD51C (H-bond) |
| L146F | VCV000410559 | RAD51C (H-bond) |
| L149R | VCV000940416 | Predicted monomer instability |
| L150P | VCV001508779 | Predicted monomer instability |
| L150R | VCV000232889 | Predicted monomer instability |
| L164P | VCV000185310 | RAD51C (H-bond) |
| L164V | VCV000239399 | RAD51C (H-bond) |
| I167F | VCV001744637 | RAD51C (H-bond) |
| V169M | VCV001745092 | RAD51C (H-bond) |
| H171Y | VCV000484776 | RAD51C (H-bond) |
| D206V | VCV001752043 | ATP (RAD51D, H-bond) |
| D206H | VCV001006029 | ATP (RAD51D, H-bond) |
| V211E | VCV001338118 | Predicted monomer instability |
| R232L | VCV000826728 | XRCC2 (H-bond) |
| R232P | VCV000581983 | XRCC2 (H-bond) |
| R232Q | VCV000138872 | XRCC2 (H-bond) |
| L234P | VCV001013719 | Predicted monomer instability |
| K235N | VCV001483803 | XRCC2 (H-bond) |
| K235M | VCV000482194 | XRCC2 (H-bond) |
| R239L | VCV000953350 | XRCC2 (H-bond) |
| R239Q | VCV000229883 | XRCC2 (H-bond) |
| R239G | VCV000472620 | XRCC2 (H-bond) |
| R239W | VCV000187225 | XRCC2 (H-bond) |
| R259P | VCV001757474 | XRCC2 (H-bond) |
| L264P | VCV000630555 | Predicted monomer instability |
| S267F | VCV000419184 | XRCC2 (H-bond) |
| F270L | VCV001762039 | XRCC2 (H-bond) |
| F270S | VCV001041684 | XRCC2 (H-bond) |
| L278P | VCV000568973 | Predicted monomer instability |
| S287Y | VCV001761631 | XRCC2 (H-bond) |
| K297Q | VCV000480545 | ATP (XRCC2, H-bond) |
| S299F | VCV000926859 | XRCC2 (H-bond) |
| R300L | VCV001692735 | ATP (XRCC2, H-bond) |
| R300Q | VCV000239405 | XRCC2 (H-bond, ionic), ATP (XRCC2, H-bond) |
| Q301H | VCV001055667 | ATP (XRCC2, H-bond) |
| P302L | VCV000539858 | ATP (XRCC2, Non-bonded) |
| T303R | VCV000919534 | ATP (XRCC2, Non-bonded) |
| T303I | VCV000823000 | ATP (XRCC2, Non-bonded) |
| T303A | VCV000187065 | ATP (XRCC2, Non-bonded) |
| I311M | VCV000322623 | RAD51D (ATP, Non-bonded) |
| I311T | VCV000127897 | RAD51D (ATP, Non-bonded) |
| I311N | VCV000127896 | RAD51D (ATP, Non-bonded) |
| I311L | VCV000960865 | RAD51D (ATP, Non-bonded) |
| I311V | VCV000584560 | RAD51D (ATP, Non-bonded) |
| **XRCC2** | | |
| I27T | VCV001492969 | Predicted monomer instability |
| I43N | VCV000596292 | Predicted monomer instability |
| G51E | VCV000658623 | ATP (XRCC2, H-bond) |
| G51R | VCV001774394 | ATP (XRCC2, H-bond) |
| T52K | VCV001337438 | ATP (XRCC2, H-bond) |
| G53A | VCV001775888 | ATP (XRCC2, H-bond) |
| K54E | VCV001480453 | ATP (XRCC2, H-bond) |
| Y59C | VCV000820044 | Predicted monomer instability |
| L61P | VCV000182992 | Predicted monomer instability |
| L79S | VCV001692897 | Predicted monomer instability |
| F80S | VCV001790692 | Predicted monomer instability |
| I81T | VCV000240160 | Predicted monomer instability |
| T83K | VCV001792040 | RAD51D (H-bond) |
| T83I | VCV000658164 | RAD51D (H-bond) |
| D84N | VCV000821397 | RAD51D (Ionic) |
| Y85S | VCV000182993 | RAD51D (H-bond) |
| D88Y | VCV000420975 | RAD51D (H-bond, ionic) |
| R91L | VCV000821725 | ATP (XRCC2, Non-bonded) |
| R91Q | VCV000486726 | ATP (XRCC2, Non-bonded) |
| R91W | VCV000182994 | ATP (XRCC2, Non-bonded) |
| F115C | VCV000823787 | RAD51D (H-bond) |
| F115L | VCV001713721 | RAD51D (H-bond) |
| Y119H | VCV001732694 | Predicted monomer instability |
| I147N | VCV000824871 | Predicted monomer instability |
| R159H | VCV000127957 | ssDNA binding |
| R159C | VCV000127956 | ssDNA binding |
| N161S | VCV000420480 | RAD51C (H-bond) |
| L183P | VCV001747850 | Predicted monomer instability |
| V190D | VCV001749136 | Predicted monomer instability |
| E274D | VCV001762529 | ATP (XRCC2, H-bond) |
| E274A | VCV001762493 | ATP (XRCC2, H-bond) |
| E274G | VCV000419716 | ATP (XRCC2, H-bond) |

**Supplementary Data Figure 1: Uncropped blots and gels**
